# Supplementary material for: Predictive validity in middle childhood of short tests of early childhood development used in large scale studies compared to the Bayley-III, the Family Care Indicators, height-for-age, and stunting: A longitudinal study in Bogota, Colombia
Source: PLoS One. 2020 Apr 29;15(4):e0231317. doi: 10.1371/journal.pone.0231317 (PMC7190101; doi:10.1371/journal.pone.0231317)
Supplement: S3 Table — (DOCX) [file pone.0231317.s003.docx]

**S4 Table. Test of Significance (*P* values) of Correlations between Age Groups: Cognitive and Language Scales of the Bayley-III, the Short Tests, the FCI, Height-for-Age, and Stunting on Enrollment with Later FSIQ and School Achievement.**

|  | **Young (6-18 Months) and Middle (19-30 Months) Age Groups** | | **Middle (19-30 Months) and Old (31-42 Months) Age Groups** | | **Young (6-18 Months) and Old (31-42 Months) Age Groups** | |
| --- | --- | --- | --- | --- | --- | --- |
| **Tests at Enrollment (6-42 months)** | **FSIQ** | **Achievement** | **FSIQ** | **Achievement** | **FSIQ** | **Achievement** |
| **Bayley-III** |  | |  | |  | |
| Cognitive | **0.026** | **0.028** | **0.038** | >0.05 | **0.000** | **0.000** |
| Receptive language | **0.019** | **0.010** | >0.05 | >0.05 | **0.000** | **0.001** |
| Expressive language | **0.023** | **0.005** | >0.05 | >0.05 | **0.000** | **0.000** |
| **ASQ-3 (adapted)** |  | |  | |  | |
| Problem solving | **0.003** | >0.05 | **0.000** | **0.004** | >0.05 | **0.005** |
| Communication | >0.05 | >0.05 | >0.05 | >0.05 | >0.05 | >0.05 |
| **Denver-II** |  | |  | |  | |
| Language | >0.05 | **0.003** | >0.05 | >0.05 | **0.001** | **0.000** |
| Fine motor-adaptive | >0.05 | >0.05 | **0.026** | **0.016** | **0.001** | **0.001** |
| **BDI-2 (Battelle)** |  | |  | |  | |
| Cognitive | >0.05 | >0.05 | >0.05 | >0.05 | >0.05 | >0.05 |
| Communication | >0.05 | **0.028** | >0.05 | >0.05 | >0.05 | **0.030** |
| **SFI & SFII (MacArthur)** |  | |  |  |  | |
| Expressive language | **0.036** | **0.027** | - | - | - | - |
| **FCI, Height-for-age, Stunting** |  | |  | |  | |
| FCI | **0.014** | **0.006** | >0.05 | >0.05 | >0.05 | >0.05 |
| Height-for-age | >0.05 | >0.05 | >0.05 | >0.05 | >0.05 | >0.05 |
| Stunting | >0.05 | >0.05 | >0.05 | >0.05 | >0.05 | >0.05 |

Number of observations for each correlation as in Table 3. ‘Young’ (6-18 months), ‘Middle’ (19-30 months), and ‘Old’ (31-42 months) age groups defined on the basis of age at enrollment. *P* values <=0.05 in bold. *P* values computed using bootstrap methods, stratifying by the design strata: socio-economic sector and age (n =2,000 replications). FCI includes play materials and play activities. Stunting is defined as height-for age <-2 SD of the WHO reference median.
