# Supplementary figures and images for: Predictive validity in middle childhood of short tests of early childhood development used in large scale studies compared to the Bayley-III, the Family Care Indicators, height-for-age, and stunting: A longitudinal study in Bogota, Colombia
Source: PLoS One. 2020 Apr 29;15(4):e0231317. doi: 10.1371/journal.pone.0231317 (PMC7190101; doi:10.1371/journal.pone.0231317)

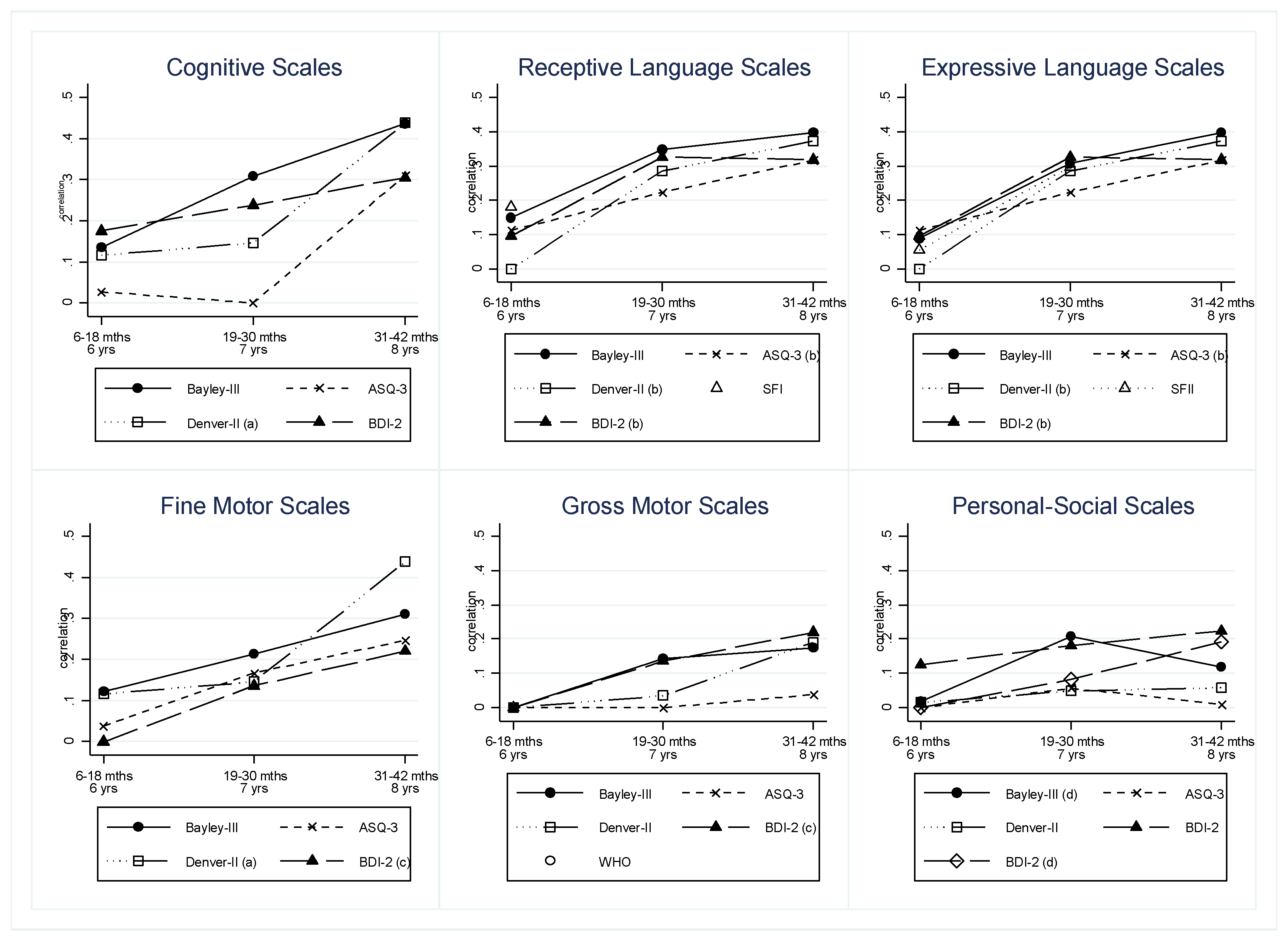

Supplement: S1 Fig — (TIF) [file pone.0231317.s004.tif]
