# Supplementary material for: Predictive validity in middle childhood of short tests of early childhood development used in large scale studies compared to the Bayley-III, the Family Care Indicators, height-for-age, and stunting: A longitudinal study in Bogota, Colombia
Source: PLoS One. 2020 Apr 29;15(4):e0231317. doi: 10.1371/journal.pone.0231317 (PMC7190101; doi:10.1371/journal.pone.0231317)
Supplement: S1 File — (PDF) [file pone.0231317.s005.pdf]

STROBE Statement—Checklist of items that should be included in reports of ***cohort studies***

	Item No	Recommendation	Page No
**Title and abstract**	1	(*a*) Indicate the study's design with a commonly used term in the title or the abstract	Title<br>Abstract: p. 1, line 21 (Background)<br>"longitudinal" study
		(*b*) Provide in the abstract an informative and balanced summary of what was done and what was found	Abstract, lines 21-38
**Introduction**			
Background/rationale	2	Explain the scientific background and rationale for the investigation being reported	Introduction, lines 49-66
Objectives	3	State specific objectives, including any prespecified hypotheses	Introduction, lines 94-108; 124-130 (objectives) and lines 116-122 (hypotheses)
**Methods**			
Study design	4	Present key elements of study design early in the paper	Methods (lines 131-144) and Fig 1
Setting	5	Describe the setting, locations, and relevant dates, including periods of recruitment, exposure, follow-up, and data collection	Methods (lines 133-154) and Fig 1
Participants	6	(*a*) Give the eligibility criteria, and the sources and methods of selection of participants. Describe methods of follow-up	Methods (lines 133-147) and Fig 1
		(*b*) For matched studies, give matching criteria and number of exposed and unexposed	NA
Variables	7	Clearly define all outcomes, exposures, predictors, potential confounders, and effect modifiers. Give diagnostic criteria, if applicable	Methods (lines 168-253) and Fig 1<br>Outcomes: lines 220-245<br>Predictors: lines 168-253
Data sources/ measurement	8*	For each variable of interest, give sources of data and details of methods of assessment (measurement). Describe comparability of assessment methods if there is more than one group	Methods<br>Outcomes: lines 220-245<br>Predictors: lines 168-253
Bias	9	Describe any efforts to address potential sources of bias	Methods, lines 267-270
Study size	10	Explain how the study size was arrived at	Methods, lines 133-147
Quantitative variables	11	Explain how quantitative variables were handled in the analyses. If applicable, describe which groupings were chosen and why	Methods, lines 258-266 and lines 271-288
Statistical methods	12	(*a*) Describe all statistical methods, including those used to control for confounding	Methods, lines 267-320
		(*b*) Describe any methods used to examine subgroups and interactions	Methods, lines 314-317
		(*c*) Explain how missing data were addressed	Not applicable for main variables in analysis. For some explanatory variables, see footnote Tables 1 and 2
		(*d*) If applicable, explain how loss to follow-up was addressed	Methods, lines 267-270

		(*e*) Describe any sensitivity analyses	Methods, robustness tests, lines 450-454
**Results**			
Participants	13*	(a) Report numbers of individuals at each stage of study—eg numbers potentially eligible, examined for eligibility, confirmed eligible, included in the study, completing follow-up, and analysed	Results (lines 314-323) and Figure 1
		(b) Give reasons for non-participation at each stage	Results (lines 316-318) and Figure 1
		(c) Consider use of a flow diagram	Figure 1
Descriptive data	14*	(a) Give characteristics of study participants (eg demographic, clinical, social) and information on exposures and potential confounders	Results (lines 323-332) and Table 1
		(b) Indicate number of participants with missing data for each variable of interest	Table 1
		(c) Summarise follow-up time (eg, average and total amount)	NA
Outcome data	15*	Report numbers of outcome events or summary measures over time	Table 1
Main results	16	(*a*) Give unadjusted estimates and, if applicable, confounder-adjusted estimates and their precision (eg, 95% confidence interval). Make clear which confounders were adjusted for and why they were included	Results, Table 3 (correlation analysis)
		(*b*) Report category boundaries when continuous variables were categorized	NA
		(*c*) If relevant, consider translating estimates of relative risk into absolute risk for a meaningful time period	NA
Other analyses	17	Report other analyses done—eg analyses of subgroups and interactions, and sensitivity analyses	Results, robustness tests, lines 450-454
**Discussion**			
Key results	18	Summarise key results with reference to study objectives	Discussion, lines 456-484
Limitations	19	Discuss limitations of the study, taking into account sources of potential bias or imprecision. Discuss both direction and magnitude of any potential bias	Discussion, lines 547-561
Interpretation	20	Give a cautious overall interpretation of results considering objectives, limitations, multiplicity of analyses, results from similar studies, and other relevant evidence	Discussion, lines 486-546
Generalisability	21	Discuss the generalisability (external validity) of the study results	Discussion, lines 529-546
**Other information**			
Funding	22	Give the source of funding and the role of the funders for the present study and, if applicable, for the original study on which the present article is based	Financial Disclosure

*Give information separately for exposed and unexposed groups.

Note: An Explanation and Elaboration article discusses each checklist item and gives methodological background and published examples of transparent reporting. The STROBE checklist is best used in conjunction with this article (freely available on the Web sites of PLoS Medicine at http://www.plosmedicine.org/, Annals of Internal Medicine at http://www.annals.org/, and Epidemiology at http://www.epidem.com/). Information on the STROBE Initiative is available at http://www.strobe-statement.org.
