# Supplementary material for: Predictive validity in middle childhood of short tests of early childhood development used in large scale studies compared to the Bayley-III, the Family Care Indicators, height-for-age, and stunting: A longitudinal study in Bogota, Colombia
Source: PLoS One. 2020 Apr 29;15(4):e0231317. doi: 10.1371/journal.pone.0231317 (PMC7190101; doi:10.1371/journal.pone.0231317)
Supplement: S2 File — Study protocols approved by IRB at Instituto de Ortopedia Infantil Roosevelt in Bogota, Colombia (in Spanish). (PDF) [file pone.0231317.s006.pdf]

Midiendo el desarrollo de niños y niñas latino-americanos y estimando la brecha socio-económica seguimiento a 5 años y medio

Marta Rubio-Codina, Banco Inter-Americano de Desarrollo, Washington DC (Investigador Principal)
M Caridad Araujo, Banco Inter-Americano de Desarrollo, Washington DC (co-Investigador)
Orazio Attanasio, Institute for Fiscal Studies, Londres (co-Investigador)
Paula Bernal (consultor, co-investigador)
Sally Grantham-McGregor, Institute for Child Health (consultor, co-Investigador)
Andrea Solano (consultor, co-Investigador)

Organizaciones Involucradas

Banco Inter-Americano de Desarrollo (BID) y Institute for Fiscal Studies (IFS)

Nota para el Comité:

El estudio que se presenta a continuación corresponde a la segunda fase de un proyecto sobre medición del desarrollo infantil temprano, que inició en el 2011 y cuyos protocolos fueron revisados por el Comité de Ética en Investigaciones del Instituto Roosevelt (IN-2015-007).

1. Introducción

Comprender el proceso de formación de habilidades (cognitivas, de lenguaje, motoras, socio-emocionales) y de acumulación de capital humano, así como el impacto de intervenciones y programas de promoción del desarrollo infantil en la formación y acumulación de dichas habilidades en el corto, mediano y largo plazo, se ha convertido en una prioridad en el mundo académico y en las agendas de política pública. Un área clave para el desarrollo de estas agendas es el contar con instrumentos que permitan la medición del Desarrollo Infantil Temprano (DIT) a gran escala en poblaciones dispares (barrios con altos niveles de marginalidad en grandes ciudades, zonas rurales remotas, etc.) de forma costo-efectiva. La falta de pruebas de desarrollo que cumplan con estos requisitos, especialmente para niños menores de 3 años, es un importante factor que limita muchas evaluaciones, así como el diseño de intervenciones.

Si bien existe un número (limitado) de instrumentos de diagnóstico para la medición de las diferentes dimensiones del desarrollo en niños pequeños, su administración puede resultar muy costosa debido a que requiere de profesionales especializados y altamente entrenados, y/o de condiciones controladas (ser administradas en un espacio cerrado y tranquilo, sin distracciones). Por ejemplo, las Escalas de Desarrollo Infantil de Bayley (Bayley Scales of Infant and Toddler Development, Bayley 1969; 1993), una prueba internacionalmente reconocida y ampliamente usada para la medición del desarrollo infantil en bebés y niños menores de 42 meses (Fernald et al. 2009; Frongillo et al. 2014), dura 90 minutos, en promedio, y requiere de materiales específicos y costosos. Por ejemplo, el kit de administración cuesta $1200 US y se requiere el pago de una licencia de alrededor de $4 US por niño a quien se administra la prueba. Además, este instrumento debe ser administrado por un profesional del desarrollo infantil (psicólogo infantil, pediatra, terapeuta infantil, etc.) luego de haber recibido un entrenamiento largo y riguroso, de semanas, que incluya la práctica suficiente para garantizar una administración adecuada y estandarizada.

Existen también pruebas de menor longitud y más fácil administración, tales como instrumentos de tamizaje y/o de reporte materno. No obstante, poco se sabe sobre su validez en entornos distintos a aquellos para los que fueron diseñadas (por lo general, poblaciones representativas de países de ingreso alto, como los Estados Unidos), así como su capacidad de medición del DIT de forma confiable cuando se administran a escala en situaciones no-controladas y cuando la administración está a cargo de personal no especializado—es decir, en situaciones similares a las de las evaluaciones de intervenciones y políticas públicas, viables tanto desde un punto de vista operativo como presupuestario.

En el 2011 recolectamos una serie de indicadores del DIT en una muestra representativa de niños y niñas de entre 6 y 42 meses en estratos 1 a 3 en Bogotá usando (i) la tercera edición del Bayley Scales of Infant and Toddler Development (Bayley-III, Bayley 2006) y (ii) una batería de pruebas más cortas y de más fácil administración *('pruebas cortas' de ahora en adelante)*. Tres de ellas eran pruebas de tamizaje que miden múltiples dimensiones del desarrollo—Ages and Stages Questionnaires (ASQ-3, Squires et al. 2009), Denver Developmental Screening (Denver-II, Frankenburg et al. 1990; Frankenburg et al. 1992), Battelle Developmental

Inventory screener (BDI-2, Newborg 2005)—y las otras dos eran pruebas que miden una única dimensión del desarrollo—los inventarios cortos de MacArthur-Bates Short-Forms (SFI y SFII, Jackson-Maldonado et al. 2012) y los Hitos del Desarrollo Motor de la OMS (WHO-Motor, WHO 2006). A fin de replicar un procedimiento de administración susceptible de ser reproducido a gran escala, las pruebas cortas se administraron en el hogar del niño, en presencia de la madre[1], por encuestadoras (sin experiencia previa en administración de pruebas ni nociones de psicología) luego de recibir una capacitación rigurosa e intensa. En la misma sesión en la que se administran las pruebas cortas, se administra también una encuesta de hogar que recaba información socio-demográfica básica. Por su parte, la prueba de Bayley-III se administró en condiciones 'ideales'—en un centro por una psicóloga—una semana después de la administración de la batería de pruebas cortas.

Este estudio, y segunda fase de este proyecto, propone medir el desarrollo cognitivo, de lenguaje y socio-emocional, así como el desempeño escolar de los niños de la muestra de Bogotá durante el segundo semestre de 2016, cuando tengan entre 6 y 9 años, con el objetivo de evaluar la validez predictiva relativa del Bayley-III y las pruebas cortas administradas en la primera fase del estudio (a los 6-42 meses).

La administración de estas pruebas se realizará en la escuela a la que asista el niño o niña. Previa a la medición en las escuelas, se hará una visita a los hogares de los niños y niñas de la muestra para administrar a la madre, padre o responsable, una breve encuesta de hogar que indague acerca de la trayectoria educativa—en la primera infancia, preescolar y escolar—del niño o niña, el nivel de estimulación en el hogar y otros aspectos socio-demográficos básicos.

Esta segunda medición permitirá establecer la **validez predictiva de desarrollo futuro y desempeño escolar de las pruebas cortas en relación al Bayley-III** que fueron administrados en la primera fase, cuando los niños tenían entre 6 y 42 meses de edad, por área de desarrollo y por grupo etario. Esto permitirá evaluar la habilidad relativa de las pruebas cortas para medir indicadores relevantes educativos y de desarrollo en la niñez y contribuirá notablemente al debate sobre qué indicadores utilizar en la evaluación de intervenciones de DIT en la primera infancia.

Asimismo, los datos de este nuevo operativo contribuirán al estudio de la **evolución en el tiempo de la brecha socio-económica** por área del desarrollo y por edad, así como al de aquellos **factores que contribuyen a cerrar diferencias socio-económicas en desarrollo y en rendimiento escolar**. Se considerarán tanto factores en el hogar como, y muy particularmente, factores en ***el entorno educativo*** durante la primera infancia, el preescolar, la pre-primaria y la primaria. Este trabajo complementará los estudios desarrollados y publicados con los datos del primer levantamiento sobre brechas socio-económicas y factores mediadores de estas brechas (Rubio-Codina et al. 2015; Rubio-Codina et al. 2016a).

[1] A lo largo del documento, usaremos la palabra 'madre' para referirnos a la madre, padre o cuidador principal del niño indistintamente.

2. Justificación

En 2013, se estimaba que más de 200 millones de niños menores de cinco años en países de ingreso bajo y medio no estaban alcanzando el desarrollo total de su potencial, dadas las condiciones de vida en las que se encontraban inmersos: pobreza, enfermedades, desnutrición, falta de higiene, falta de oportunidades educativas, entornos familiares desestructurados, violentos, y en general poco responsivos y afectuosos, entre otras condiciones negativas (Grantham-McGregor et al. 2007). Ello genera retrasos en el DIT muy difíciles de remediar en edades más avanzadas dada la plasticidad del cerebro en los primeros años de vida (Shonkoff 2010).

Varios estudios empíricos—ver Almond y Currie (2010) para un compendio—muestran cómo las consecuencias de la exposición a estos factores de riesgo en la edad temprana se mantienen a lo largo de la vida del individuo—traduciéndose en mayor deserción escolar, menor rendimiento académico, menos oportunidades laborales, peor salud física y psíquica en la adolescencia y la edad adulta, y peor calidad de vida en general. Esto supone no sólo un costo para el individuo sino también para la sociedad (Naudeau et al. 2011). Al mismo tiempo, existe un número creciente de estudios clínicos y pilotos a pequeña escala que muestran el potencial que tienen las intervenciones en la primera infancia sobre la reducción de retrasos en DIT en el corto plazo, así como sus consecuencias en el mediano y largo plazo (Engle et al. 2011).

Trasladar estas experiencias a intervenciones y programas de política bien diseñados, adecuadamente focalizados y costo-efectivos es una necesidad imperante, que requiere como paso previo el **avance de la agenda de medición del DIT** tanto para la identificación de áreas prioritarias de intervención como para la evaluación del impacto de las políticas diseñadas. Es decir, requiere de la identificación de instrumentos de medición de fácil y rápida administración, que sigan siendo válidos y confiables cuando se usen a escala. Es preciso que estos instrumentos sean buenos predictores de indicadores de interés—entre otros, el desarrollo cognitivo, de lenguaje, y socio-emocional en la edad escolar, el desempeño académico, aspectos conductuales, regulatorios y de función ejecutiva, que a su vez estén correlacionados con el éxito personal y profesional y el bienestar en la edad escolar, en la adolescencia y en la edad adulta.

Varias organizaciones, incluyendo la Organización Mundial de la Salud, El Instituto Brookings, El Banco Mundial y la UNESCO, se encuentran en la actualidad desarrollando indicadores de desarrollo infantil temprano (DIT) para uso a escala. Existen también esfuerzos similares en la comunidad académica. Medir el DIT a nivel poblacional y evaluar los pilotos, intervenciones y programas de promoción del DIT en curso con **indicadores costo-efectivos y predictivos del desarrollo futuro es una necesidad preponderante para conseguir que el DIT alcance la relevancia política que requiere y para movilizar el financiamiento que demanda.**

Los datos de Bogotá son únicos. No conocemos de ningún otro estudio que cuente con información sobre DIT de tan alta calidad, medido con tantos instrumentos distintos, para una muestra de niños tan pequeños y representativa de hogares de ingreso bajo y medio. Es por

ello que tiene el potencial de contribuir de forma notable a los esfuerzos de medición del DIT en curso a nivel internacional.

3. Problema y Pregunta de Investigación

La medición del desarrollo infantil a edades tempranas es compleja y costosa: las pocas pruebas de diagnóstico existentes toman más de una hora para su administración; requieren de personal especializado y específicamente formado; de un espacio que facilite la concentración del niño; y generalmente no han estado validadas en contextos distintos a aquellos para las que fueron diseñadas. Estos factores son un obstáculo importante para su uso tanto en la evaluación de programas sociales como en la identificación de áreas óptimas de intervención. Dados los importantes retornos de invertir en la primera infancia tanto a nivel individual como poblacional, es de primera necesidad identificar instrumentos alternativos que permitan la medición del DIT a escala y de forma costo-efectiva. Asimismo, es importante establecer hasta qué punto estos instrumentos, administrados en condiciones factibles a escala, son predictivos de aquellos indicadores (escolaridad, desempeño académico, desarrollo futuro, etc.) de interés y relevantes para la política pública.

Las preguntas específicas de investigación son:

1. ¿Cuál es la validez predictiva relativa de las pruebas administradas a los 6-42 meses (pruebas cortas entre sí y con respecto al Bayley-III) del desempeño académico y del desarrollo cognitivo, de lenguaje y socio-emocional 5.5 años más tarde? ¿Qué pruebas cortas o qué sub-escalas son un mejor reflejo del desarrollo en la edad escolar y del desempeño académico a nivel global y por dimensión del desarrollo? Todo teniendo en cuenta que las pruebas cortas se administraron en el hogar por personal no especializado, como ocurre frecuentemente en la evaluación de programas e intervenciones.

   Las preguntas secundarias relacionadas con esta primera pregunta general son:

   1.1. ¿Existen diferencias en la validez predictiva por grupo etario o por dimensión del desarrollo?
   1.2. ¿Existen diferencias en la validez predictiva por nivel socio-económico de los hogares?
   1.3. Dados los resultados sobre validez predictiva (objeto de este estudio) y los hallazgos sobre confiabilidad, validez concurrente y costos en la administración de las pruebas en base a los datos recabados en la primera fase de este estudio (Rubio-Codina et al 2016b), ¿cuál o cuáles serían las pruebas cortas más costo-eficientes para la evaluación de programas sociales y/o de promoción del DIT? ¿Qué elementos se deben tener en cuenta en la decisión sobre qué prueba administrar?

2. ¿Cuál es el tamaño de la brecha en desarrollo y aprovechamiento escolar a los 6-9 años de edad por dimensión del desarrollo y por grupo etario? ¿Es estadísticamente significativa?

   Las preguntas secundarias relacionadas con la segunda pregunta general son:

2.1. ¿En qué medida la brecha socio-económica a los 6-9 años de edad está determinada por el tamaño de la brecha prexistente en la primera infancia?
2.2. ¿Qué factores son mediadores y moderadores de dicha brecha socio-económica? ¿Qué rol juegan los centros de cuidado e instituciones educativas durante la primera infancia, la edad preescolar y la edad escolar? ¿Qué papel juega la educación parental, así como el entorno en el hogar?
2.3. ¿Existen diferencias por género tanto en la magnitud y evolución de la brecha socio-económica, como en el papel de factores mediares y moderadores?

4. Marco Teórico

Este estudio se enfoca en la validez predictiva de pruebas administradas a niños de 6 a 42 meses ya que, es en este grupo etario, donde la medición del desarrollo infantil es más compleja y el vacío de instrumentos es mayor. Para niños mayores de 3 años existen más pruebas de medición del DIT, así como más estudios que abordan el tema de la medición y/o documentan gradientes socio-económicos en DIT.

En relación a estudios que comparan el desempeño de pruebas cortas administradas a escala por personal encuestador, así como su validez concurrente, cabe destacar el trabajo de Hamadani y colegas en zonas rurales de Bangladesh para las áreas de motricidad gruesa y de lenguaje. En particular, Hamadani et al. (2013) investigan la validez concurrente entre la edad de logro de hitos de desarrollo motor—medido por personal encuestador y por reporte materno—con el índice de desarrollo psicomotor del Bayley-II (PDI) en una muestra de cerca de 2.000 niños. Los autores hallaron una correlación moderada, especialmente entre los hitos pararse solo y caminar solo, así como una capacidad predictiva de desarrollo futuro de estos hitos tan alta como el PDI, tanto para la medición del encuestador como para el reporte materno. De forma similar, Hamadani et al. (2010) desarrollaron una prueba de medición de lenguaje (lista de vocabulario) por reporte materno para niños de 12 a 18 meses basada en el MacArthur Communicative Development Inventories que ofrece validez concurrente moderada con el índice de desarrollo mental del Bayley-II y una validez predictiva aceptable de coeficiente intelectual a los 63 meses de edad medido con el Wechsler Preschool and Primary Scale of Intelligence (WPPSI).

Como referente de la medición del desarrollo cognitivo, y en particular, del coeficiente escolar, tomamos la versión más reciente (quinta) de la prueba de *Wechsler Intelligence Scale for Children* (WISC-V), considerada como la prueba de referencia (patrón de oro) a nivel internacional para el diagnóstico (Weschler 2014). Para el desempeño escolar tomamos las sub-escalas relevantes en la ***Batería-III Woodcock-Muñoz: Pruebas de Aprovechamiento*** (Woodcock and Johnson 1977; Muñoz-Sandoval et al 2005a, b), la cual ha sido ampliamente usada en América Latina.

5. Objetivos

5.1. General

Este proyecto tiene dos objetivos generales diferenciados:

1. Establecer la validez predictiva de desarrollo y desempeño escolar 5.5 años más tarde de las pruebas cortas y el Bayley-III por dominio, grupo etario y nivel socio-económico del hogar del niño.

2. Cuantificar la magnitud de las brechas existentes en desarrollo en la edad escolar y en desempeño escolar para niños de 6-9 años de edad en una gran urbe representativa de otras grandes ciudades en países de ingreso medio y medio-bajo.

5.2. Específicos

Dados los dos objetivos generales anteriores, los objetivos específicos de este estudio son:

1.1. Establecer si/qué pruebas de desarrollo son de administración suficientemente costo-efectiva para uso en evaluaciones de intervenciones de DIT y de programas sociales a escala, así como para seguimientos poblacionales, teniendo en cuenta los criterios de: viabilidad de uso (costos de compra, capacitación y administración), confiabilidad, validez concurrente y validez predictiva. Esta segunda fase del proyecto contribuye específicamente a la validez predictiva.

1.2. Identificar y discutir los factores determinantes (objetivos de la medición, edades de los niños, dimensiones del desarrollo, disponibilidad de recursos, validez concurrente y predictiva) en la elección de pruebas para la medición del DIT.

2.1. Identificar factores mediadores y moderadores del gradiente socio-económico en desarrollo en la edad escolar y desempeño académico por dimensión del desarrollo.

2.2. Identificar la persistencia de brechas iniciales, así como el rol de factores mediadores y moderadores.

2.3. Identificar si existen diferencias por género tanto en el tamaño y evolución de la brecha, como en el rol de factores mediadores y moderadores.

2.4. Informar el diseño de intervenciones y programas de promoción del DIT y de servicios educativos.

6. Metodología

6.1. Diseño de la investigación

El diseño del estudio es no experimental. En la primera fase del estudio se recogió un corte transversal de una muestra representativa de niños y niñas por grupos de edad (de 6 a 42 meses) y estrato socio-económico (estratos 1 a 3) en la ciudad de Bogotá, Colombia.

6.2. Hipótesis

Las *hipótesis conceptuales* del estudio, de naturaleza *correlacional*, relacionadas con el objetivo 1 son:

H1.1. La validez predictiva es mayor entre dominios coincidentes (por ejemplo, entre sub-escalas de lenguaje que entre una sub-escala de lenguaje y una sub-escala cognitiva).

H1.2. La validez predictiva es creciente con la edad del niño en la primera medición.

H1.3. La validez predictiva por dominio del desarrollo y por grupo etario es mayor en hogares de mayor nivel socio-económico (asociados a madres con mayor nivel educativo) que en hogares de menor nivel socio-económico (asociados a madres con menor nivel educativo).

Las *hipótesis conceptuales* del estudio relacionadas con el objetivo 2 son:

H2.1. Los niños en hogares con mayor nivel socio-económico muestran mayores niveles de desempeño académico y de desarrollo en la edad escolar que los niños en hogares con menor nivel socio-económico. Estas diferencias son estadísticamente significativas en promedio.

H2.2. El mayor nivel educativo de los padres, la presencia de los padres en el hogar y la calidad del ambiente del hogar del niño en términos psicosociales (tiempo con el niño, acceso a materiales de juego, receptividad parental, etc.) contribuyen a la reducción de las brechas socio-económicas; es decir, son factores mediadores.

H2.3. La asistencia a centros de cuidado y la escolarización contribuyen significativamente a cerrar brechas socio-económicas; es decir, son factores mediadores.

Las hipótesis H1.1. y H1.3. son de naturaleza *correlacional*; la hipótesis H1.2. es de *diferencia de grupos*.

Las *hipótesis nulas* del estudio negarían lo establecido en las hipótesis conceptuales.

6.3. Población y Muestra

6.3.1. Población Diana

La muestra de estudio incluye los 1.330 niños y niñas en la muestra de Bogotá a los cuales se les administró la prueba de Bayley-III en 2011.[2] Estos niños habitan en hogares de estratos 1 a 3 en la ciudad de Bogotá y tenían entre 6 y 42 meses durante la recolección de la primera ronda de datos entre marzo y agosto de 2011. En el momento de esta nueva medición, estos niños tendrán entre 6 y 9 años.

6.3.2. Criterios de Selección de la Muestra

Al tratarse de un estudio de seguimiento, los **criterios de inclusión** son todos los niños en la muestra de 1,330 con medición de Bayley-III. No hay criterio de exclusión. Tampoco se permiten los reemplazos.

[2] La muestra completa de Bogotá incluye 1.533 hogares, pero sólo se pudo administrar la prueba de Bayley-III en 1.330 niños en estos hogares. Estos 1.330 niños y sus hogares son los que se incluyen en este segundo seguimiento.

6.3.3. Muestreo

La selección de la muestra original fue por diseño probabilístico a partir del conteo (censal) de niños de 6 a 42 meses en todos los hogares en las manzanas (bloques) incluidas en la muestra por selección aleatoria. La muestra se estratificó por grupo etario y por nivel socio-económico.

La distribución de niños y manzanas (bloques) por grupo etario y estratos es la siguiente:

Tabla 1: Número de niños y manzanas (bloques) en la muestra de Bogotá por grupo etario (en el momento de la selección) y estrato socio-económico en 2011

Edad	Estrato 1	Estrato 2	Estrato 3	Estrato 4	Total
**6-14 meses**	108	104	100	2	314
**15-23 meses**	87	112	133	0	332
**24-32 meses**	103	125	117	7	352
**33-41 meses**	105	118	107	2	332
**Total niños**	403	459	457	11	1330
**Niños por manzana**	3,0	2,9	2,3	2,2	2,7
**Total manzanas**	134	159	199	5	497

6.3.4 Tamaño de la Muestra

Como actividades preparatorias, se ejecutó durante el 2015 un operativo de ubicación de los hogares de la muestra, vía correo electrónico, llamadas telefónicas y visitas a los hogares. Los resultados muestran niveles de pérdida relativamente bajos (entorno al 84%), dada la alta movilidad en poblaciones urbanas de bajo estrato. Además, el 76% de los hogares en la muestra, mostró interés en seguir participando en el estudio. Se prevé un nuevo operativo de actualización de muestra, que recupere al 16% de los hogares que no se encontraron durante el operativo de ubicación de 2015, así como aquellos hogares que hayan migrado desde dicho operativo, además de volver a contactar e instar a participar a aquellos hogares que manifestaron no estar interesados en dar continuidad a su participación en el estudio. Estas acciones se harán con el fin de maximizar la participación en el estudio del número total de hogares de la muestra original—siempre y cuando la no participación no se deba a causas de fuerza mayor (por ejemplo, defunción).

Los tamaños de muestra esperados permitirían identificar diferencias alrededor de 0,3 desviación estándar (DS) de un puntaje estandarizado (con media 1 y DS igual a 0) por estrato-grupo y grupo de edad (6-18 meses, 19-30 meses y 31-42 meses) al 80% de poder y nivel de significancia del 5%. Este orden de magnitud es similar o menor al impacto estimado de intervenciones de promoción del DIT vía visitas domiciliarias en países como Bangladesh (Nahar et al. 2012) y Jamaica (Grantham-McGregor et al. 1991), cuyo objetivo es cerrar brechas socio-económicas.

6.4. Instrumentos, Recolección de la información

El operativo de campo se realizará en 3 etapas:

1. **Operativo de ubicación, a cargo del equipo de actualización**. Este equipo estará a cargo de llamar a los hogares de la muestra para validar la información de contacto y proceder a entregar los registros al equipo encuestador que será el responsable de aplicar la encuesta en el hogar. Asimismo, este equipo de actualización contactará telefónicamente al 16% de los hogares que no se ubicaron en el operativo de 2015. Si el contacto telefónico no es logrado a través de este operativo, los registros serán entregados al equipo encuestador para que las direcciones con que se cuenta sean visitadas en campo. Durante el contacto con los hogares ubicados, el miembro del equipo de actualización deberá explicar el contenido del estudio y recordar o informar a quien atienda, que hubo una primera medición 5.5 años atrás, y generar en los hogares interés y voluntad de participar nuevamente.

2. **Encuesta de hogar a cargo del equipo encuestador que se realizará en el hogar del niño**. Una encuestadora visitará el hogar para la administración de un breve cuestionario, con una duración aproximada de 45 minutos. Este cuestionario será respondido por la madre, padre, responsable legal/tutor, o cuidador principal del niño objeto de estudio. Recabará información básica sobre el hogar y sus miembros, con particular foco en el niño objeto del estudio. Incluirá también información sobre la calidad del ambiente en el hogar y el historial de asistencia a centros de cuidado infantil y escolarización del niño objeto de estudio. Incluirá también un instrumento de temperamento y conducta, administrado por reporte materno, el *Strengths and Difficulties Questionnaire* (SDQ, Goodman, R.1999). Será imprescindible recabar el número del documento de identificación de la mamá, del papá o del responsable legal, así como del niño, y el nombre de la institución educativa a la que el niño asiste en la actualidad. Se adjunta un primer borrador de la encuesta de hogar (Anexo I).

   La administración de la encuesta de hogar tendrá lugar inmediatamente después a la firma del consentimiento informado para protección de datos. Este consentimiento explícitamente solicitará la participación del niño en el estudio y en particular en la evaluación psicométrica que el evaluador (psicólogo) desarrollará en la institución educativa como parte de la tercera etapa del estudio, descrita a continuación.

   Para atraer a hogares renuentes se plantea la posibilidad de hacer una rifa de cien mil pesos m/cte ($100.000) por cada 100 hogares, donde se haya logrado completar la encuesta de hogar y la aplicación de pruebas psicométricas con los niños. Este incentivo servirá para apoyar la aplicación de pruebas, luego de completar el diligenciamiento de la encuesta de hogar, y se prevé que ayude a mitigar el rechazo a la participación en el estudio.

3. **Evaluación psicométrica a cargo del equipo evaluador (psicólogos) que se realizará en la institución educativa del niño**. Un evaluador (psicólogo) administrará una serie de pruebas psicométricas al niño en una sesión de no más de 1 hora y 30 minutos (75 minutos de evaluación + 15 minutos de descansos intercalados).

Durante esta sesión se administrarán algunas las siguientes pruebas:

(i) Las 7 sub-pruebas de la quinta versión de la *Wechsler Intelligence Scale for Children* (WISC-V) que constituyen **el Full Scale IQ (coeficiente intelectual o CI**). El WISC es una de las tres pruebas en las Escalas de Weschler para la medición de inteligencia o coeficiente intelectual, considerado el 'patrón de oro' en la medición de esta capacidad. El WISC-V está diseñado para ser aplicado en niños de 6 a 16 años de edad, inclusive, y su administración toma entre 50 y 75 minutos (110 minutos si se administra la prueba completa).[3] El WISC se ha usado en muchos países, incluyendo países de habla hispana tales como Venezuela y México (Fernald et al. 2009).

(ii) Sub-escalas relevantes en el WISC-V para la para la medición de **aprovechamiento escolar** (habilidades matemáticas y de lenguaje/lectura) y función ejecutiva.

(iii) Sub-escalas relevantes en la Batería-III Woodcock-Muñoz: Pruebas de Aprovechamiento (Woodcock and Johnson 1977; Muñoz-Sandoval et al 2005a, b) para la medición de **aprovechamiento escolar** (habilidades matemáticas y de lenguaje/lectura) y función ejecutiva. Esta batería incluye 22 pruebas que miden cinco áreas curriculares: lectura, expresión oral, expresión escrita, matemática y conocimiento académico, por administración directa sobre el niño. Ha sido ampliamente usada en América Latina y se han establecido normas para poblaciones de habla hispana usando muestras de varios países en la región y el conteniente, incluyendo Colombia, así como una muestra de población hispana en Estados Unidos (Schrank et al. 2005).

(iv) Una versión adaptada de la versión en español del **Peabody Picture Vocabulary Test (PPVT)**, o **Test de Vocabulario en Imágenes de Peabody (TVIP)** (Dunn et al. 1986) para la medición del lenguaje. El TVIP mide el nivel vocabulario el niño o niña a partir del reconocimiento de imágenes que representan o reflejan la palabra estímulo mencionada por el evaluador. Ha sido ampliamente usado en América Latina (Fernald et al. 2009; Schady et al. 2015).

(v) Pruebas que midan la función ejecutiva. Se está considerando el Card Sorting (Zelazo, 2006) y varias pruebas de Stroop (Jensen and Rowher 1966; MacLeod 1991; Stroop 1935) adaptadas. La función ejecutiva es un conjunto de habilidades de autorregulación que incluyen: el control inhibitorio, la memoria de trabajo, la flexibilidad cognitiva y la atención, todas estas centrales en el proceso de aprendizaje (Anderson 2002; Espy 2004; Senn et al. 2004). Las pruebas que se están considerando miden especialmente el control inhibitorio y la flexibilidad cognitiva. Las habilidades que hacen parte de la función

[3] http://www.pearsonclinical.com/psychology/form/field-examiner.html

ejecutiva tales como el autocontrol, son buenos predictores de las trayectorias de aprendizaje y del éxito de una persona en el largo plazo, incluyendo resultados en el mercado laboral (Moffitt et al. 2011; Séguin and Zelazo 2005).

La decisión final sobre qué pruebas y sub-pruebas aplicar se tomará con base en un pre-piloto que estará liderado por el equipo investigador, incluyendo el psicólogo líder y un psicólogo sénior, en calidad de asesor técnico.

Se redactará una carta para el personal de las instituciones educativas, firmada por una autoridad local del sector educativo que respalde el estudio y genere credibilidad en la comunidad, con el objetivo de facilitar la cooperación durante el trabajo en campo.

La administración de la evaluación psicométrica tendrá lugar después dela encuesta de hogar. El lapso de tiempo podrá llegar a ser de hasta 2 meses, permitiendo así el procesamiento de la información sobre las instituciones educativas a las que asisten los niños (información recabada en el hogar) y la organización del operativo en dichas instituciones lo más eficiente posible (es decir, que optimice tiempos y desplazamientos). No obstante, en ningún caso se podrá evaluar a los niños antes de que cumplan los 6 años. Asimismo, se deberá asegurar que tanto el equipo encuestador como evaluador recabarán información de los niños en todos los estratos y a lo largo de todo el rango etario de interés.

Equipo de campo y capacitación

El desarrollo de este estudio requiere contar con un equipo de evaluadores alta y rigurosamente formados para la aplicación de los diferentes test psicométricos.

La recolección de toda la información en el hogar (etapa 1) está a cargo de 16 encuestadoras con amplia experiencia en recolección de encuestas, quienes serán capacitadas durante 1 semana en la administración del cuestionario de hogar. Trabajarán en grupos de 4 por zona, con una supervisora en cada grupo. La capacitación del equipo encuestador estará a cargo de personal especializado en la empresa de datos con la que trabajamos.

Todas las actividades de evaluación en la institución educativa (etapa 2) están a cargo de psicólogas con especialización o maestría, quienes recibirán una capacitación de 5 semanas de duración en la administración de la pruebas psicométricas a administrar al niño. Se prevé que aproximadamente el 60% del tiempo de capacitación se va a destinar a prácticas de campo. La capacitación del equipo de psicólogos estará a cargo de la psicóloga líder del proyecto, bajo la tutela de la psicóloga senior. Amplias psicólogas cuentan con amplia experiencia en la administración de pruebas de evaluación del desarrollo infantil y en la capacitación de las mismas.

Adecuación y pilotaje de las pruebas

Los formatos de las pruebas psicométricas no se adjuntan por dos motivos: (i) para no violar derechos de autor asociados a las mismas y (ii) porque todavía no se han definido las versiones finales de los mismos (ni las pruebas que finalmente se administrarán como parte del estudio). Esto se definirá en base a las actividades de pilotaje. En cualquier caso, las pruebas a administrar serán pruebas estandarizadas que serán traducidas (en los casos que sea necesario) y cuyo contenido (expresiones, palabras, imágenes) será adecuado al contexto para garantizar la comprensión lingüística y funcional de la prueba. El objetivo del piloto es precisamente asegurar que estas traducciones y adaptaciones son adecuadas y estará a cargo de la psicóloga líder, bajo la mentoría de la psicóloga sénior.

6.5. Variables

Las principales variables de estudio son las siguientes [en corchetes se incluyen los instrumentos, pendientes de ser piloteados]:

Variables dependientes - nivel de desarrollo de niño:
(La lista de pruebas finales se definirá en función del resultado de los pilotajes)

- Desarrollo cognitivo, coeficiente intelectual [WISC-V, Full Scale IQ]
- Desarrollo de lenguaje (expresivo y receptivo) [TVIP, sub-escalas del WISC-V y de la Batería-III de WM]
- Desarrollo socio-emocional [SDQ]
- Función ejecutiva [sub-escalas relevantes del WISC-V y de la Batería-III de WM, Card Sorting, Stroop test]

Variables explicativas:
(Dado que se trata de un seguimiento, no se recabará información invariante en el tiempo con respecto a la primera medición).

- Factores biológicos del niño [lactancia exclusiva y destete]
- Educación de los padres [años de escolaridad de la madre y el padre]
- Nivel de riqueza del hogar [activos, características de la vivienda, estrato]
- Composición demográfica del hogar [número de hermanos, miembros del hogar y edades]
- Calidad del ambiente del hogar [versión adaptada del HOME-Middle Childhood]
- Escolaridad [asistencia a la escuela, tipo de centro, costo]
- Histórico de asistencia a centros de cuidado y educativos [asistencia a centros de cuidado, prescolar y primaria por tipo corriente e histórico]

6.6. Calidad del dato, control de sesgos y error

Con el fin de maximizar la calidad de la información recolectada y minimizar el error de medición y posibles sesgos en los datos, se tomarán las siguientes medidas:

- Traducción, adaptación y/o contextualización de los instrumentos al uso del español en Colombia y al contexto; y pilotaje de las nuevas versiones y/o adaptaciones—a cargo de la psicóloga que lidera la capacitación.
- Aplicación de 2 administraciones en un mismo niño con una semana de diferencia en al menos 10 niños para cada prueba para el cálculo de la *confiabilidad de test-retest*

(correlación intra-clase, ICC), que garantice la estabilidad en el tiempo del instrumento traducido y/o adaptado/contextualizado (ICC mayor a 0,7).

- *Rigurosa capacitación de aproximadamente 5 semanas, incluyendo prácticas* para el equipo de psicólogos, con el objetivo de alcanzar fluidez y rigurosidad en la recolección de la información, así como estandarizar la administración de las pruebas. Como parte de la capacitación, se requerirán entre 15 y 20 prácticas por persona y prueba, a ser desarrolladas en parejas. Durante estas prácticas se recabará la confiabilidad tanto entre-evaluadoras (entre parejas de evaluadoras psicólogas) como con el capacitador. Se continuará practicando hasta alcanzar ICCs mayores a 0,9. Estas prácticas se realizarán con niños en fundaciones y escuelas privadas y de la Secretaría de Educación del Distrito en niños en estratos y dentro del rango etario de los niños en la muestra.
- *Supervisión y calificación de 5% de las administraciones en campo* por parte de la capacitadora psicóloga para el cálculo de la confiabilidad entre evaluadora y la capacitadora a lo largo del operativo. Esta supervisión estará repartida de igual forma entre todas las evaluadoras, así como por edad del niño y por nivel socio-económico del hogar al que pertenecen.
- *Fiel monitoreo del avance del operativo* en campo tal que se garantice que:
    - Todas las evaluadoras evalúan la misma proporción de niños de diferentes edades
    - Todas las evaluadoras evalúan la misma proporción de niños pertenecientes a diferentes niveles socio-económicos (estratos)
    - La evaluación de niños de diferentes edades y pertenecientes a diferentes niveles socio-económicos está balanceada a lo largo del tiempo que dura el operativo.

6.7. Plan de análisis de la información

Plan de análisis para preguntas relacionadas con el objetivo 1

1. Construcción de puntajes crudos (continuos) para todas las sub-escalas de todas las pruebas siguiendo las indicaciones en los manuales de las mismas.
2. Estandarización interna de los puntajes crudos tal que se elimine el efecto de la edad y del evaluador/entrevistador y se facilite la comparación entre las pruebas.
3. Construcción puntajes de Full Scale IQ, lenguaje, aprovechamiento escolar (matemática y lenguaje) y función ejecutiva, según los resultados de un análisis factorial exploratorio sobre los puntajes crudos de cada sub-prueba.
4. Para cada sub-escala en cada prueba corta, cálculo de la validez predictiva entre dicha sub-escala con la sub-escala correspondiente en la prueba de Bayley-III usando el coeficiente de correlación de Pearson; total y por edad.
5. Cálculo de correlaciones entre sub-escalas no coincidentes; totales y por edad.
6. Repetir cálculos de validez predictiva en los puntos 4 y 5 por nivel socio-económico del hogar.
7. Análisis de mínimos cuadrados ordinarios:

$$Y_i = \beta_0 + \beta_1\overrightarrow{X_i} + \beta_3 Y_{i,t-1} + \varepsilon_i$$

Dónde:

Y_i : Puntaje en la segunda medición

$Y_{i,t-1}$: Puntaje en la primera medición

$\vec{X}$: Vector de características del niño. Incluye: género, edad, educación de la madre y estatus nutricional del niño

8. Determinar si las diferencias en las correlaciones por (i) grupos etarios; (ii) nivel socio-económico del hogar; y (iii) entre pruebas, son estadísticamente significativas usando métodos de bootstrap para el cálculo de los intervalos de confianza.
9. Cálculo del coeficiente de consistencia interna (agregado y por grupo de edad).

Plan de análisis para preguntas relacionadas con el objetivo 2

1. Estandarización interna de los puntajes crudos tal que se elimine el efecto de la edad y del evaluador/entrevistador y se facilite la comparación entre las pruebas.
2. Construcción puntajes de Full Scale IQ, lenguaje, aprovechamiento escolar (matemática y lenguaje) y función ejecutiva, según los resultados de un análisis factorial exploratorio sobre los puntajes crudos de cada sub-prueba.
3. Cálculo de un índice de riqueza usando características de los hogares y tenencia de activos por el método de componentes principales usando correlaciones policóricas. Construcción de cuartiles y quintiles de la distribución normalizada de este índice.
4. Estimación del coeficiente por cuartil del índice socio-económico sobre el desarrollo del niño por sub-escala, a través de una regresión (por Mínimos Cuadrados Ordinarios con efecto de conglomerado a nivel sección) que adicionalmente controla por la edad y género del niño. Considerando el primer cuartil o quintil como referencia, el coeficiente sobre el último cuartil o quintil corresponde a la diferencia entre el 25% más rico y el 25% más pobre en la muestra en términos de desarrollo infantil, o gradiente socio-económico del desarrollo. Análisis para todas las observaciones para las que se cuente con información y por grupo etario.
5. Controlar en la regresión por el impacto de otros factores explicativos (biológicos, características parentales, calidad del ambiente en el hogar, asistencia al prescolar, escuela y duración, etc.) para estudiar el potencial efecto mediador de estos factores en el tamaño de la brecha socio-económica.

6.8 Consideraciones Éticas

En base a la resolución No. 008430 de 1993 del Ministerio de Salud de la República de Colombia, el presente estudio tiene categoría de *investigación en seres humanos de riesgo mínimo* dado que es prospectivo y emplea el registro de datos a través de procedimientos comunes, en este caso pruebas psicológicas a individuos, mediciones de peso y talla, y una encuesta (Artículo 11). En ningún caso se pretende manipular la conducta del sujeto previa ni posterior a la administración de las pruebas psicológicas, encuesta o toma de mediciones antropométricas (Artículo 11). La mayoría de las pruebas se administra directamente sobre el niño, aunque algunas, como el SDQ, es por reporte materno.

La logística y procedimientos operativos de capacitación y recogida de información estarán a cargo de una empresa de recolección de datos con sede en Bogotá y con experiencia previa este tipo de operativos, *Sistemas Especializados de Información* SEI S.A. Todas las actividades de diseño de la muestra, capacitación y levantamiento de información estuvieron

rigurosamente definidas junto con los miembros del equipo de investigación, quienes las supervisaron de forma constante.

El protocolo de recogida de la información cumple con las siguientes consideraciones éticas:

- Toda recolección de información tendrá lugar luego de la firma del Consentimiento Informado por escrito por parte del sujeto (en el caso de la madre o cuidador principal) o del representante legal (en el caso del niño) (Artículo 15). Dicho consentimiento garantiza el compromiso de confidencialidad de la información tanto en su obtención como en su manejo, así como su uso para fines exclusivamente de investigación, garantizando de este modo el derecho a la privacidad (Artículo 8). Asimismo, el consentimiento aclara que el participante puede cancelar la administración y retirarse del estudio en cualquier momento, garantizando así la dignidad de los participantes y la protección de sus derechos y bienestar (Artículo 5, título II, capítulo 1). El Consentimiento Informado se incluye en el Anexo II en la documentación adjunta.
- La evaluación y las actividades asociadas a las mismas serán realizadas por profesionales con conocimiento y experiencia, procurando en todo momento el bienestar de los participantes (Artículo 6f.). Dichos profesionales, del perfil profesional estipulado en los manuales de administración de las pruebas, serán capacitados debidamente y hasta que su administración sea adecuada y esté convenientemente estandarizada. Además seguirán rigurosamente los protocolos de investigación establecidos. Se revisará también su Certificado Judicial previo a la capacitación.
- Los niños que manifiesten retrasos en su desarrollo, en base a los umbrales establecidos por la prueba de WISC-V, se derivarán al centro de consultas especializado en atención a niños con problemas de desarrollo más cercano a la residencia del niño y su familia. Las psicólogas prepararán los formatos de remisión a usar.

Siguiendo los requisitos de la Ley de Protección de Datos Habeas Data, el protocolo de almacenamiento y procesamiento de la información a seguir cumplirá con las siguientes consideraciones éticas:

- Todos los datos procesados estarán **anonimizados** y serán almacenados en discos protegidos por contraseñas.
- Las bases con nombres estarán guardadas en un disco duro de acceso altamente restringido en el IFS, al que sólo tiene acceso la investigadora principal del proyecto.

Beneficios para los participantes en el estudio

La medición del desarrollo a través de la administración de las varias pruebas no es una actividad que cause ningún daño ni al niño ni a su familia. Por el contrario, la experiencia puede ser un espacio positivo de interacción y juego para el niño. Al final de la administración se enviará a la madre información acerca del desempeño de su hijo. Este documento será redactado de forma muy neutral y constructiva por parte de la psicóloga líder y la psicóloga senior, quien cuenta con mucha experiencia en la evaluación de niños de estas edades y en la comunicación con sus padres.

Consideraciones éticas para las prácticas

Las prácticas se realizaron con niños de fundaciones, escuelas públicas y privadas de la ciudad. Con previa aprobación del rector de la institución y firma del consentimiento informado por parte de los padres. En agradecimiento se ofrecerá a estas instituciones:

1. Un taller para los profesionales del centro para dar a conocer en términos generales los resultados encontrados con respecto al desarrollo cognitivo de los niños evaluados (bajo entendido que la prueba no está normada para Colombia y que las administraciones se hicieron por profesionales en periodo de prácticas y por lo tanto, el resultado no debía ser considerado como definitivo), así como posibles estrategias de intervención.
2. Los resultados de las pruebas a las familias bajo los mismos entendidos y reservas que en el punto 1, según la preferencia de los miembros de la institución educativa.

7. Administración del proyecto

7.1 Recursos

El Banco Inter-Americano de Desarrollo (BID) ha aportado $200,282 US para financiar el operativo de campo y todas las actividades asociadas, incluyendo los costos de obtención, adaptación y capacitación de las pruebas y los honorarios de los psicólogos responsables.

El detalle del presupuesto se muestra a continuación:

	TOTAL COP	TOTAL USD
**Costes Profesionales - consultorías**		**$ 12,839**
Psicóloga líder - capacitadora 7 meses	$ 28,750,000	$ 10,268
Psicóloga sénior	$ 7,200,000	$ 2,571
**Viajes equipo de investigación y otros costs (comité ético)**	**$ 15,000,000**	**$ 5,357**
**Pruebas psicométricas**		**$ 33,514**
WISC V		$ 19,450
Bateria III WM - materiales asociados a escalas relevantes		$ 8,420
Peabody Spanish - formatos administración		$ 2,444
Costos otras pruebas función ejecutiva (estimado)		$ 3,200
**Operativo de campo**		**$ 148,571**
Cotización SEI	$ 416,000,000	**$ 148,571**
**GRAND TOTAL (x-rate at 2800)**		**$ 200,282**

El tiempo de los investigadores asociados al proyecto se financió con recursos propios (de las instituciones a los que están asociados los investigadores).

7.2 Cronograma

La siguiente tabla muestra el cronograma del proyecto:

**Fechas**	**Actividad**
Mayo – Junio 2016	*Inicio actividades*<br>– Identificación equipo de trabajo (psicólogos, empresa datos), definición instrumentos, organización y preparación de actividades<br>- Contratación psicóloga a cargo de la adecuación y capacitación de las pruebas de desarrollo<br>- Contratación empresa de datos

	- Compra y obtención de pruebas.<br>-Tramitación licencias – casas editoriales
Junio – Julio 2016	*Actividades relacionadas con las pruebas*<br>- Familiarización de los psicólogos capacitadores con las pruebas, incluyendo administración de las mismas<br>- Actividades de adecuación de pruebas y 'pre-pilotaje'<br>- Diseño del operativo con empresa de datos<br>- Diseño, pilotaje y diagramación encuesta de hogar
Julio – Agosto 2016	- Convocatoria y selección de encuestadores<br>- Capacitación encuestadores<br>- Actividades de actualización de muestra
Agosto – Noviembre 2016	*Operativo de campo muestreo y encuestas:*<br>1. Organización operativo en escuelas<br>2. Gestionar espacios en escuelas y citas<br>3. Preparar materiales finales para capacitación y preparar capacitación<br>4. Convocatoria evaluadoras<br>5. Selección evaluadoras<br>6. Capacitación evaluadoras<br>7. Prácticas de campo<br>8. Operativo evaluadoras – medición en escuelas<br>9. Supervisión campo
Noviembre – Diciembre 2016	-Sistematización de información en bases y chequeo de inconsistencias por parte de SEI, S.A.
23 Diciembre 2016	-Entrega de bases al equipo investigador
Enero – Febrero 2017	-Revisión de las bases y chequeos de inconsistencias por parte del equipo investigación. Depuración de las bases y entrega de las bases finales.
Marzo 2017	-Pegue de datos con la primera medición, limpieza datos; construcción de puntajes crudos para todas las pruebas; construcción de variables socio-económicas; cálculo de confiabilidades durante las prácticas y supervisión campo.
Abril – Junio 2017	*Actividades de análisis y documentación de resultados*

8. Resultados Esperados

8.1. Fortalecimiento de la apropiación social del conocimiento

Los resultados del presente estudio serán relevantes para profesionales y hacedores de política pública interesados en la medición del DIT a escala y en el diseño y evaluación de intervenciones que lo promuevan. El estudio tiene como objetivo generar evidencia empírica acerca de:

- El perfil del desarrollo cognitivo, de lenguaje y socio-emocional, así como el desempeño escolar, en la etapa escolar primaria y la evolución de la brecha en DIT por dimensión del desarrollo para una población representativa de clase baja y media de entre 6 y 9 años.

- El efecto (persistencia) de las brechas en desarrollo en la primera infancia en las brechas en desarrollo en la edad escolar.
- Los factores parentales, de estimulación en el hogar e institucionales que contribuyen a cerrar o agrandar estas brechas.
- La capacidad de medición del desarrollo futuro (validez predictiva) de pruebas cortas de más fácil administración al ser administradas por personal encuestador capacitado en el hogar del niño, por edad y área de desarrollo en relación a la prueba de Bayley-III.

Por su naturaleza—la medición del DIT con tantos instrumentos distintos, para una muestra de niños tan pequeños y representativa de hogares de ingreso bajo y medio—este estudio tiene el potencial de contribuir de forma notable a los esfuerzos de medición del DIT en curso a nivel internacional.

8.2. Fortalecimiento de la comunidad científica

Los resultados del estudio descritos en el punto anterior serán también relevantes para investigadores y miembros de la comunidad científica y académica, en particular en el área social. Generarán recomendaciones sobre el tipo de instrumento a usar para la medición de DIT en la evaluación de políticas sociales y de DIT a escala y/o en situaciones de recursos limitados. Dado el auge e importancia que el DIT ha adquirido en los últimos años, existe una necesidad cada vez mayor de identificar instrumentos que permitan su medición de forma costo-efectiva.

9. Transferencia de Resultados

Los resultados serán diseminados a través de presentaciones formales al gobierno de Colombia y a otros gobiernos e instituciones interesadas en la región, contrapartes del BID. Asimismo, estos resultados se presentarán en círculos académicos e institucionales tanto en seminarios académicos como en talleres de trabajo, instituciones de acogida de los investigadores (BID, IFS) foros nacionales, regionales e internacionales.

El equipo de investigación trabajará también en la producción de artículos de divulgación científica a ser publicados en revistas académicas de revisión de pares (peer-review) de alto nivel. Se ha publicado ya dos artículos en revistas en ciencias sociales con un proceso de revisión de pares externo con los datos de la primera fase de este estudio (Rubio-Codina et al. 2015 y Rubio-Codina et al. 2016a) y hay un tercer artículo en proceso de revisión en una revista médica (Rubio-Codina et al. 2016b). Se prevé publicar también los resultados de esta nueva medición. Asimismo, se escribirán también versiones de las investigaciones en formatos distintos—documentos de trabajo, reportes técnicos, reportes divulgativos, entre otros—de diferente formato y nivel de detalle, para garantizar que los resultados de la investigación lleguen a todas las potenciales audiencias: comunidad científica, hacedores, ejecutores o evaluadores de política pública e intervenciones y donantes.

Toda publicación hará mención al Comité de Ética del Instituto de Ortopedia Infantil Roosevelt.

10. Bibliografía

Almond, Douglas, and Janet Currie. 2010. "Human Capital Development before Age Five." In *Handbook of Labor Economics*, ed. Orley Ashenfelter and David Card, 4b(15):1315-1486. Elsevier B.V.

Anderson, P.J. 2002. "Assessment and Development of Executive Functioning (EF) in Childhood." *Child Neuropsychology* 8(2): 71-82.

Attanasio Orazio P, Fernández Camila, Fitzsimons Emla O A, Grantham-McGregor Sally M, Meghir Costas, Rubio-Codina Marta. 2014. "Using the infrastructure of a conditional cash transfer program to deliver a scalable integrated early child development program in Colombia: cluster randomized controlled trial". *British Medical Journal* 2014; 349:g5785

Bayley, Nancy. 2006. *Bayley Scales of Infant and Toddler Development – Third Edition*, San Antonio, Texas: Harcourt Assessment.

Bayley, Nancy. 1969. *Manual for the Bayley scales of infant development.* San Antonio, TX: The Psychological Corporation.

Bayley, Nancy. 1993. *Bayley scales of infant development—Second edition.* San Antonio, TX: The Psychological Corporation

Espy, K.A. 2004. "Using Developmental, Cognitive, and Neuroscience Approaches to Understand Executive Functions in Preschool Children." *Developmental Neuropsychology* 26(1): 379-84.

Fernald, C. H. Lia, Patricia Kariger, Melissa Hidrobo, and Paul J. Gertler. 2012. "Socioeconomic Gradients in Child Development in Very Young Children: Evidence from India, Indonesia, Peru, and Senegal." *Proceedings of the National Academy of Sciences* 109(2):17273-80.

Fernald, C. H. Lia, Patricia Kariger, Patrice Engle, and Abbie Raikes. 2009. "Examining Early Child Development in Low-Income Countries: A Toolkit for the Assessment of Children in the First Five Years of Life." The World Bank, Washington D.C.

Edward A. Frongillo, Fahmida Tofail, Jena D. Hamadani, Andrea M. Warren and Syeda F. Mehrin. 2014. "Measures and indicators for assessing impact of interventions integrating nutrition, health, and early childhood development", *Annals of the New York Academy of Sciences*, 1308: 68-88: DOI: 10.1111/nyas.12319

Engle, P.L., Fernald, L.C., Alderman, H., Behrman, J., O'Gara, C., Yousafzai, A., Cabral de Mello, M., Hidrobo, M., Ulker, N., Ertem, I., Iltus, S. and the Global Child Development Steering Group. 2011. Strategies for reducing inequalities and improving developmental outcomes for young children in low-income and middle-income countries. *The Lancet* 378(9799):1339-1353.

Hamadani J.D., Tofail, F., Huda, S.N., Alam, D.S., Ridout, D.A., Attanasio, O. and Grantham-Grantham-McGregor Sally, Yin B. Cheung, Santiago Cueto, Paul Glewwe, Linda Richter, Barbara Strupp, and the International Child Development Steering Group. 2007. "Developmental Potential in the First 5 Years for Children in Developing Countries." *The Lancet* 369(9555):60-70.

Goodman, R. (1999). The extended version of the Strengths and Difficulties Questionnaire as a guide to child psychiatric caseness and consequent burden.*Journal of child psychology and psychiatry*, *40*(05), 791-799.

Grantham-McGregor S, Powell C, Walker S, Himes J. 1991. Nutritional supplementation, psychosocial stimulation, and mental development of stunted children: the Jamaican Study. *Lancet* 1991;338:1-5.

Jensen, A.R., and W.D. Rowher. 1966. "The Stroop Color-Word Test: A Review." *Acta Psychologica* 25(1): 36-93.

MacLeod, C.M. "Half a Century of Research on the Stroop Effect: An Integrative Review." *Psychological Bulletin* 109(2): 163-203.

Macours, Karen, Norbert Schady, and Renos Vakis. 2012. "Cash Transfers, Behavioral Changes, and Cognitive Development in Early Childhood: Evidence from a Randomized Experiment." *American Economic Journal: Applied Economics* 4(2):247-73.

Moffitt, T., L. Arseneault, D. Belsky, N. Dickson, R. Hancox, H. Harrington, R. Houts, R. Poulton, B. Roberts, S. Ross, M. Sears, M, Thomson, and A. Caspi. 2011. "A Gradient of Childhood Self-Control Predicts Health, Wealth, and Public Safety." *Proceedings of the National Academy of Sciences* 108(7): 2693-98.

Nahar B, Hossain MI, Hamadani JD, Ahmed T, Huda SN, Grantham-McGregor SM, 2012. "Effects of a community-based approach of food and psychosocial stimulation on growth and development of severely malnourished children in Bangladesh: a randomised trial". *Eur J Clin Nutr*; 66:701-9

Naudeau, Sophie, Sebastian Martinez, Patrick Premand, and Deon Filmer. 2011. "Cognitive Development among Young Children in Low-Income Countries." In *No Small Matter. The impact of Poverty, Shocks, and Human Capital Investments in Early Childhood Development*, ed. Harold H. Alderman. The World Bank, Washington D.C.

Paxson, Christina, and Norbert Schady. 2007. "Cognitive Development among Young Children in Ecuador: The Roles of Wealth, Health, and Parenting." *Journal of Human Resources* 42(1):49-84.

Rubio-Codina M, Attanasio OP, Meghir C, Varela N, Grantham-McGregor SM. 2015. "The Socio-Economic Gradient of Child Development Children 6-42 Months in Bogota", *Journal of Human Resources* 50(2):464-483.

Rubio-Codina M, Attanasio OP, Grantham-McGregor SM. 2016a. "Mediating Pathways in the Socio-Economic Gradient of Child Development: Evidence from Children 6-42 Months in Bogota", forthcoming at the *International Journal of Behavioral Development*.

Rubio-Codina M, Araujo C, Attanasio OP, Muñoz P, Grantham-McGregor SM. 2016b. Concurrent Validity and Feasibility of Short Tests Currently Used to Measure Early Childhood Development in Large Scale Studies, revise and resubmit to *PlosOne*.

Schady, N., Behrman, J., Araujo, M. C., Azuero, R., Bernal, R., Bravo, D., ... & Vakis, R. (2015). Wealth gradients in early childhood cognitive development in five Latin American countries. *Journal of Human Resources*, *50*(2), 446-463.

Séguin, J., and P. Zelazo. 2005. "Executive Function in Early Physical Aggression." In R. Tremblay, V. Hartup, and J. Archer, Eds., *Developmental Origins of Aggression* (pp. 307-29). New York: The Guilford Press.

Senn, T.E., K.A. Espy, and P.M. Kaufmann. 2004. "Using Path Analysis to Understand Executive Function Organization in Preschool Children." *Developmental Neuropsychology* 26(1): 445-64.

Shonkoff, P. Jack. 2010. "Building a New Biodevelopmental Framework to Guide the Future of Early Childhood Policy." *Child Development* 81(1):357-367.

Stroop, R. 1935. "Studies of Inference in Serial Verbal Reactions." *Journal of Experimental Psychology* 18(6): 643-662.

Diamond, A., Taylor, C. 1996. Development of an aspect of executive control: Development of the abilities to remember what I said and to do as I say, not as I do. *Developmental Psychobiology, 29,* 315-334.

Dunnn, Ll., E. Padilla, D. Lugo and L. Dunn. (1986). *Manual del Examinador para el Test de Vocabulario en Imágenes Peabody (Peabody Picture Vocabulary Test), Adaptación Hipanoamericana (Hispanic-American Adaption)*, Pearson Assessments, Minneapolis.

Dunnn, Ll. and L. Dunn. (1981). *Peabody Picture Vocabulary Test-Revised*, Dunn Educational Services, Inc., Diablo, California
Harms, T., Clifford, R. M., & Cryer, D. (1998). Early Childhood Environment Rating Scale. New York: Teachers College Press.

Harms, T., Cryer, D., & Clifford, R. (2003). Infant and Toddler Environment Rating Scale Revised. New York: Teachers College Press.

Muñoz-Sandoval, A. F., Woodcock, R. W., McGrew, K. S. and Mather, N. (2005a). *Batería III Woodcock-Muñoz*. Itasca, IL: Riverside Publishing.

Muñoz-Sandoval, A. F., Woodcock, R. W., McGrew, K. S. and Mather, N. (2005b). *Batería III Woodcock-Muñoz: Pruebas de aprovechamiento*. Itasca, IL: Riverside Publishing.

Muñoz-Sandoval, A. F., Woodcock, R. W., McGrew, K. S. and Mather, N. (2005c). *Batería III Woodcock-Muñoz: Pruebas de habilidades cognitivas*. Itasca, IL: Riverside Publishing.

Radloff LS. The CES-D scale: a self-report depression scale for research in the general population. *Appl Psychol Meas* 1977;1:385-401.

Wechsler, D. (2014). Wechsler Intelligence Scale for Children—Fifth Edition. San Antonio, TX: NCS Pearson.

Wechsler, D. (2004). The Wechsler intelligence scale for children—fourth edition. London: Pearson Assessment.

Wechsler, D. (1974). Manual for the Wechsler Intelligence Scale for Children—Revised. New York: Psychological Corporation.

Zelazo, P. D. (2006). The Dimensional Change Card Sort (DCCS): A method of assessing executive function in children. *NATURE PROTOCOLS-ELECTRONIC EDITION-*, *1*(1), 297.

11. Anexos

Ver carpeta con la siguiente documentación adjunta:

- Anexo I – Cuestionario Hogar
- Anexo II – Consentimiento Informado
