# Supplementary material for: Predictive validity in middle childhood of short tests of early childhood development used in large scale studies compared to the Bayley-III, the Family Care Indicators, height-for-age, and stunting: A longitudinal study in Bogota, Colombia
Source: PLoS One. 2020 Apr 29;15(4):e0231317. doi: 10.1371/journal.pone.0231317 (PMC7190101; doi:10.1371/journal.pone.0231317)
Supplement: Attachment — Submitted filename: Response to Reviewers.docx [file pone.0231317.s008.docx]

**PONE-D-19-20029**

**Predictive validity in middle childhood of short tests of early childhood development used in large scale studies compared to the Bayley-III, the Family Care Indicators, height-for-age, and stunting: A longitudinal study in Bogota, Colombia**

**Responses from the authors to the Reviewer’s comments**

We thank the Reviewers for reading through our first set of responses in detail and for their subsequent comments to continue improving the Manuscript. We respond below to the new set of reviewers’ comments. For clarity we retain the comments and add our response in *italics.* We have numbered the reviewers’ comments to facilitate cross-references to our responses. Please note that the references to the line numbers where edits have been made correspond to the version of the Manuscript without tracked changes (clean version).

**Reviewer Comments to the Author**

**Reviewer #1: PONE-D-19-20029R1
Predictive validity in middle childhood of short tests of early childhood development used in large scale studies compared to the Bayley-III, the Family Care Indicators, height-for-age, and stunting: A longitudinal study in Bogota, Colombia**

The study presents analyses of mental development data from a cohort 6 to 42 months from Bogata showing data on predictive validity. The purpose is to provide data that would allow researchers to select short measures of early child development, with evidence that they are sound alternatives to the Bayley III in predicting WISC and academic scores in middle childhood. The methodology and statistical analyses were rigorously conducted.

1. One limitation is that the findings are limited to descriptive/ longitudinal purposes; the authors cannot convincingly address program evaluation, though they would like to. We do not know how sensitive is the Denver to evaluate a nutrition-WASH-stimulation program.

*Response: This is an important limitation, but also one that is beyond the scope of this work. We have acknowledged this in lines 488-491, which have been rewritten to refer to impact evaluations of cash transfer programs using some of the short tests—the Denver-II in Nicaragua and the SFII in Ecuador— where both have been found to be sufficiently sensitive to identify intervention impacts. These lines now read: “**Whilst both the Denver-II and the SFII have been found to be sensitive to the impact of cash transfer programs in Nicaragua (Macours, Schady, & Vakis, 2012) and Ecuador (Fernald & Hidrobo, 2011), respectively, further investigation of sensitivity to interventions for all short tests would be helpful in guiding the choice of tests.” In the immediately previous sentences (lines 478-488), we had emphasized that our recommendations on test choice were dependent on the purpose of use of the test.*

2. A second limitation is that researchers will shortly have a short global measure and a longer program evaluation measure to use instead of the Denver Screening Test or other existing alternatives. Already they have the MDAT which was not included in this study and not mentioned in the ms.

*Response: Indeed, a short and long global measure for the assessment of child development, the Global Scales of Early Development or GSED, is being developed. This is exciting news for the discipline and the authors have been involved with their development. However, the GSED short and long forms will not be available for some time since both tests are still at different stages of piloting in six different countries. In addition, further piloting might be required in different areas of the world to ensure that their properties are maintained worldwide, across languages and cultures (i.e. that they are truly global measures). Furthermore, investigation of their predictive validity and response to interventions will take considerably longer to be available. The analysis performed in this work is therefore useful whilst these global measures are under development and fully validated, as noted in the Manuscript in lines 48-50 and lines 61-64.*

*As explained, the MDAT was not included in the study since it was not published when the study began and was not well known at the time (Gladstone et al., 2010). In addition, time and budgetary constraints prevented us from using and evaluating other tests. We have added the need to use other tests/further investigation to be able to extrapolate findings outside urban Latin America as a limitation in lines 506-510, which now reads: “Finally, the above findings could be extrapolated to urban Colombia and possibly urban areas in other Latin American countries. Further studies would be required before extrapolating to rural areas and other LMICs, especially in Africa and Asia, which could additionally evaluate more short tests for these contexts, such as the Malawi Developmental Assessment Tool (MDAT) (Gladstone et al., 2010).” We have also added the reasons why the MDAT and other tests had not been used in lines 510-512: “The number of short tests included in the current study was limited due to time and budgetary constraints. We therefore chose tests that were commonly used in large surveys and evaluations at the time the study began”.*

Abstract and elsewhere

3. Evaluation of interventions and predictive validity of such tests entail two separate issues. Predictive validity is the main phrase in the title; however the first sentence of the Abstract concerns the evaluation of interventions at scale. Evaluation is not really “hindered by the lack of developmental tests” because large-scale evaluations can validly assess representative samples to compare with controls. Your reply to this comment was arguably limited to purchases from the US, and did not consider the important value for LMIC in training local researchers and the value in translation and adaptation. Parent-report measures similar to those used in this study are subject to bias if the intervention concerns psychosocial stimulation: mothers who receive messages about child development now have beliefs about when developmental milestones occur and mothers who learn about interacting with their child will have more accurate observations of when milestones are attained (see WB toolkit p52). So use of the ASQ instead of a direct child assessment provides weak support. Frankly, the data here address predictive validity and not evaluations of interventions; reference to evaluation of interventions in the Introduction and Discussion should be dropped as it misguidedly encourages the use of screeners and parent reports in interventions. If you think that the Denver or another measure has been effectively used to evaluate a large unconditional cash transfer program, this can be mentioned in the Discussion as additional information about the test being advocated.

*Response: We agree with the Reviewer on the possible limitations of the use of screeners with parent reports in the evaluation of early childhood interventions and had already expressed this in lines 478-484. Furthermore, our results do not support the use of the ASQ-3, which is entirely parent report. They do support the use of the Denver-II, and in particular, the use of the language and cognitive scales of the test, which have at least 61% of the items directly administered to the child, as indicated in lines 179-182.*

*In this paragraph we also emphasize the importance of the purpose of the assessment when choosing a test (line 478) and the need for further investigation on the sensitivity to intervention of the short tests to further guide the choice of tests to use (lines 488-491). Yet, to avoid confusions we have dropped the relevant references to the use of the short tests for evaluations in the abstract (lines 18-19) and the introduction (lines 43-45) which now read, respectively: “However, progress is hindered by the lack of valid developmental tests feasible for use at large scale.” (abstract) and “However, progress of these efforts is impeded by the lack of reliable, valid, and easy-to-collect measures of ECD, particularly for children under age three years (Black et al., 2017; Fernald, Prado, Kariger, & Raikes, 2017; McCoy, Black, Daelmans, & Dua, 2016).” (introduction).*

*Thank you for the suggestion to refer to impact evaluation studies that have used any of the short tests. We have added two references that refer to impact evaluation studies that have used two of the short tests: these are the evaluation of cash transfer programs in Nicaragua (which used the Denver-II) and in Ecuador (which used the SFII) in lines 488-491, which read: “Whilst both the Denver-II and the SFII have been found to be sensitive to the impact of cash transfer programs in Nicaragua (Macours et al., 2012) and Ecuador (Fernald & Hidrobo, 2011), respectively, further investigation of sensitivity to interventions for all short tests would be helpful in guiding the choice of tests.” There is also recent data on the use of the Denver-II for evaluation in a large-scale program in China. One of the authors was involved with the study and selected the Denver-II test for evaluation, subsequent to the findings from the present study. A significant effect was found (analysis by Nobel Laureate, James Heckman) providing evidence of the Denver-II’ s sensitivity to intervention effects. Unfortunately, the paper is in preparation and presently unpublished, so we hesitate to cite it here. We cite it here only to provide information for the Reviewer and Editor.*

Introduction and Enrollment

4. Lines 51-53. This sentence should be revised: “Whilst existing diagnostic tests such as the Bayley Scales [5,6] are sensitive to ECD interventions [2,7–9], they are very expensive, take a long time to administer and require highly trained testers with a certain level of technical expertise [2].” Only in some countries are they expensive, take a long time to deliver and require highly trained testers … It might be more accurate to state, “According to our experience, …they are very expensive…” I would have to say that only in full do they take a long time, and rarely have I seen highly trained testers doing the training. I wonder how many other researchers had to spend 6 weeks training psychology graduates on the Bayley III. You might want to consider that this is your experience; colleagues in Peru had the same experience so I don’t doubt your word. But it is not a feature of the test and not necessarily the experience of others [I’m not even sure that the authors of the WB 2017 toolkit have the experience of training others on the Bayley].

*Response: We are afraid we disagree with this point, as we have met researchers in many countries who find the Bayley difficult to give and expensive. We have rewritten the sentence in lines 50-54 as suggested and it now reads “Whilst existing diagnostic tests such as the Bayley Scales (Bayley, 1969, 2006) are sensitive to ECD interventions (Attanasio et al., 2014; Fernald et al., 2017; Hamadani, Huda, Khatun, & Grantham-McGregor, 2006; Nahar et al., 2009), according to our experience and that of many other researchers, they are very expensive, take a long time to administer and require highly trained testers with a certain level of technical expertise (Fernald et al., 2017).” This has been the experience of the many researchers we have consulted, some of whom are part of the GSED and DScore teams (Cavallera et al., 2019; Weber et al., 2019), as well as authors of the WB2017 toolkit.*

Methods

5. Lines 166-181. Thank you for adding more about whether tests were direct or parent report and how many items were parent report if the test included both. Appreciate also the information about including higher items on the screeners to avoid ceiling. These are critical features of child assessment.

*Response: Thank you for suggesting it. We agree that these details add important information on the assessments used.*

6. Line 282. Thank you for elaborating on the Denver motor-adaptive scale. The original Bayley [ref 5] Performance subscale combined cognitive and fine motor, so I can see how the Denver does so.
   Nothing was said about adaptation of the measures, particularly the WISC, Bayley, BSD, Denver other than translation. Could you state that no adaptations were required?

*Response: With regards to the middle childhood assessments, the WISC-V was the only test that had to be translated. For the other tests, minor wording and phrasing adaptations had to be made after piloting to adjust the language to the local use of Spanish, as indicated. We have now clarified that no other adaptations were found necessary in lines 234-236: “**We translated the WISC-V report forms and manuals and piloted the translations. All other test materials were available in Spanish and minor wording/phrasing modifications were made, after piloting, to reflect Colombian Spanish. No other adaptations were found necessary”.*

*With respect to the enrollment assessments (Bayley-III, Denver-II, etc.), details had been provided in* *[earlier work](https://publications.iadb.org/en/publication/17155/concurrent-validity-and-feasibility-short-tests-currently-used-measure-early) (see Appendix I in pages 40-42). Owing to space restrictions and to avoid duplication, we do not add these details to the current Manuscript. We have added a paragraph explaining whether any translation/adaptation was required and have provided the reference to the earlier work containing the full details in lines 198-203: “In preparation for testing, we translated and back-translated the Bayley-III; as well as the BDI-2 manual, and the WHO-Motor report forms and manual. Short tests in battery A were all available in Spanish. All translations and official Spanish versions were piloted and, subsequently, minor wording/phrasing modifications were made in order to reflect Colombian Spanish. Similarly, a few images had to be contextualized. Full adaptation details were provided earlier (Rubio-Codina, Araujo, Attanasio, & Grantham-McGregor, 2016).”*

Results

7. Thank you for clarifying improvement in retest scores.

Thank you for providing age correlations in S2.
Table S2. p cannot be <.0000. Better to write p<.0001.

*Response: Thanks for pointing this out, we have modified the p-value in Table S2 as suggested. We have also modified the p-values in Table 4. We are glad that the other clarifications were helpful.*

8. Table 1. Internally standardized scores are not very meaningful. I can understand why you used them for analyses but they are not meaningful for readers who are usually given standardized Bayleys and raw HOME. Some of the raw scores were useful in S2 but it would be good to see raw HOME in order to compare with other studies (rather than internally standardized) and externally standardized scores for Bayley III and WISC. I noticed that the Bayley III standardized scores in the 2016 paper looked similar to US and UK means; they won’t always be this high in other regions of the world. Did you expect standardized IQ scores to drop in middle childhood?

*Response: Externally standardized WISC-V scores are already provided in Table 1. We have added Bayley-III externally standardized scores (composites) in Table S1. However, we would caution against the use of external norms for samples in populations other than the norming population.*

*In discussions with the authors of the HOME, we adapted the test substantially in order to make it more suitable for the study population. This is noted in lines 245-246: “In middle childhood, an adaptation of the Middle Childhood Home Observation for Measurement of the Environment (MC-HOME) (Bradley, Caldwell, Rock, Hamrick, & Harris, 1988; Caldwell & Bradley, 2003) was collected”. Therefore, comparisons of the HOME raw score in Bogota with the HOME raw scores in other studies might be somewhat misleading and, hence, we rather not show the HOME raw score in the Manuscript, particularly as the HOME is not one of the tests under study, but only acts as a ‘covariate’. However, in the next table, we report the HOME total raw score and by subscale for the Reviewer’s reference.*

9. For the other measures (ASQ, BSI, Denver) can you provide some central score or % above/below a cut-off? It is problematic that these tests do not allow for scores to be shown.

*Response: We had provided raw scores of all tests in* [*earlier work*](https://publications.iadb.org/en/publication/17155/concurrent-validity-and-feasibility-short-tests-currently-used-measure-early) *(Rubio-Codina, Araujo, Attanasio, & Grantham-McGregor, 2016, Table A1 in Appendix III, page 44). As noted in the Manuscript, the short tests used were mostly screeners and their mode of administration was modified, for some of the tests, so as to increase their ability to assess children along the developmental continuum. The language in some of the items had also been modified to ensure cultural appropriateness and functional equivalence (details of the adaptations are also provided in* [*earlier work*](https://publications.iadb.org/en/publication/17155/concurrent-validity-and-feasibility-short-tests-currently-used-measure-early)*, Rubio-Codina, Araujo, Attanasio, & Grantham-McGregor (2016), Appendix I, pages 40-42). For some short tests, these modifications made it difficult to interpret external norms. For example, we used 9 items (as opposed to the original 6) in the ASQ and the cut-off points in the original test were no longer applicable and could be misleading. We had no plan to use them as screeners or validate cutoff points. The study design and sample were not appropriate for this.*

*The purpose of the current paper is to assess the relative performance of the various short tests used to predict concurrent and future development against a gold standard (the Bayley-III). To this end, we think that the analyses carried out using internally standardized scores is more adequate.*

Discussion

10. Line 473. “recall bias” is probably not the right phrase to describe the bias of mothers who have attended a stimulation class. It may be more like an “observation bias” due to spending more time with their child, and a “desirability bias” due to knowing what milestones of development to expect.

*Response: Thanks for raising this point and for the suggestions. We have relabeled and explained the sources of bias in lines 478-484, which now read “Caregiver reports might be better suited for the evaluation of population-based indicators and less convenient to evaluate psychosocial stimulation programs as they may suffer from “observation” bias, if mothers in the treatment group spend more time with the child and are more aware of the process of development/achievement of milestones as a result of the intervention. Moreover, intervened mothers may be biased towards making optimistic claims of their children’s development to report on intervention success (“desirability” bias).”*

11. Line 487 etc. My previous comment re the FCI was “prevalence for several items asymptotes at a high rate at 4 months of age, and other items like counting and drawing asymptote at a low rate at 4 years. So not all items are relevant for low-income countries at all these ages.” The ages being referred to are between 4m and 4 year, not simply those two ages. If high rates asymptote at 4m it means they have hit ceiling for all older children. If rates asymptote at a low level by 4 years, it means they are at floor for many months before this. Have you assessed variation in your FCI scores for your full sample by age? Thank you for pointing out that the FCI will have different means in Africa and Asia.

*Response: This is a very interesting point, thanks for the clarification. The following two tables show the variation of the FCI scores by component (type of play material and type of play activity) by age groups. A few of the items (in italics) show little variation over age and seem to be at a ceiling (“toys for moving around”, “singing songs”, “taking child outside the home place/go for a walk”). The remaining items show a gradient with age.*

*We have reconstructed the FCI play activities and play materials score without the 3 items that do not show any variation and have correlated the new score to IQ and school achievement at ages 6-8 years by age group in early childhood. The correlations remain very similar to those previously obtained, as shown in the next table (see greyed area and text in blue). Therefore, we do not think that the potential lack of variability in certain FCI items is of concern and prefer to use the instrument as it was designed. The fact that the predictive validity of the FCI score without these items improves slightly after 19 months might be an indication that our estimates of predictive validity are conservative. We thank the review for raising this point as it has been an interesting analysis to carry out and an aspect to keep in mind in future work.*

12. Line 488. One problem with using the FCI to evaluate a psychosocial program is that it assesses stimulation but not responsive caregiving.

*Response: Thanks, we have added this in current lines 493-496, which now read: “It is free, quick (10-15 min) and easy to give, provides information on useful activities for parents (although not on responsive caregiving) and has been widely used in international surveys—most notably, UNICEF’s Multiple Indicator Cluster Surveys* (MICS-ECDI, n.d.)*.”*

13. The Denver Screening test appears to be the strongest substitute for the Bayley. It may have the same administration duration, requires caregiver report for 39% of the items, and costs less in the US. The authors did not provide information on length of training testers and adaptation required. This would help prospective researchers know what is required.

*Response: Details on training and adaptation were published earlier (Rubio-Codina, Araujo, Attanasio, & Grantham-McGregor, 2016; Rubio-Codina, Araujo, Attanasio, Muñoz, & Grantham-McGregor, 2016). There is a reference to both papers in lines 202-203 “Full adaptation details were provided earlier (Rubio-Codina, Araujo, Attanasio, & Grantham-McGregor, 2016).” and lines 210-212 “More details on the enrollment tests, their costs, and on the administration and training procedures were provided previously [10, 33]” of the current Manuscript. As noted in our response to Reviewer 1 comment 6, we have added some details on the adaptations needed for the early childhood assessments in lines 198-203. We hesitate to add any further details to avoid duplications and due to space restrictions. Please note that the Denver-II took a considerably shorter amount of time to administer than the Bayley-III (27 minutes vs. 83 minutes), as indicated in lines 204-210.*

14. Limitations. Reasons for not using the MDAT should be mentioned in the limitations. I understand that you were not aware of its launch in 2010 but readers will wonder why it was ignored given its currently popularity.

*Response: We have added these reasons as limitations in the Discussion in lines 506-512, which read: “Finally, the above findings could be extrapolated to urban Colombia and possibly urban areas in other Latin American countries. Further studies would be required before extrapolating to rural areas and other LMICs, especially in Africa and Asia, which could additionally evaluate more short tests for these contexts, such as the Malawi Developmental Assessment Tool (MDAT) (Gladstone et al., 2010). The number of short tests included in the current study was limited due to time and budgetary constraints. We therefore chose tests that were commonly used in large surveys and evaluations at the time the study began.”*

15. Another limitation is that height and stunting may be less predictive in Colombia than in Africa because of the narrow range of values, particularly in the stunted range.

*Response: Thanks. We agree with the Reviewer and believe that the point is covered in the paragraph corresponding to lines 501-505: “Furthermore, although stunting has been used as an indicator of inadequate child development globally (Black et al., 2017; Grantham-McGregor et al., 2007), the FCI was a better predictor of future overall intellectual functioning and school achievement in this population. If these findings are replicated in countries with different home environments and stunting severity and prevalence, combining both indicators would be a more effective population-based indicator.” and in the following paragraph in lines 506-512 (see response to Comment 14 above).*

**Reviewer #2:**

1. The authors have improved their manuscript and they sufficiently addressed my comments.
   For their understanding: The Bayley III is also available in Dutch with Dutch norms, and we published the differences between the US norms and the Dutch norms in PLOS One.

*Response: We are very glad to hear that we have sufficiently addressed Reviewer’s 2 comments. We were not aware that the Bayley-III is also available in Dutch and has Dutch norms and thank the Reviewer for bringing it to our attention. We have not modified the Manuscript with regards to Reviewer’s 2 comments.*

**References**

Attanasio, O. P., Fernandez, C., Fitzsimons, E. O. A., Grantham-McGregor, S. M., Meghir, C., & Rubio-Codina, M. (2014). Using the infrastructure of a conditional cash transfer program to deliver a scalable integrated early child development program in Colombia: cluster randomized controlled trial. *BMJ*, *349*(sep29 5), g5785–g5785. https://doi.org/10.1136/bmj.g5785

Bayley, N. (1969). *Bayley Scales of Infant Development*. New York: Psychological Corp.

Bayley, N. (2006). *Bayley Scales of Infant and Toddler Development–Third Edition: Technical manual.* San Antonio, TX: Harcourt Assessment.

Black, M. M., Walker, S. P., Fernald, L. C., Andersen, C. T., DiGirolamo, A. M., Lu, C., … Committee, L. E. C. D. S. S. (2017). Early childhood development coming of age: science through the life course. *The Lancet*, *389*(10064), 77–90. https://doi.org/10.1016/S0140-6736(16)31389-7

Bradley, R. H., Caldwell, B. M., Rock, S. L., Hamrick, H. M., & Harris, P. (1988). Home Observation for Measurement of the Environment: Development of a Home Inventory for use with families having children 6 to 10 years old. *Contemporary Educational Psychology*, *13*(1), 58–71. https://doi.org/10.1016/0361-476X(88)90006-9

Caldwell, B. M., & Bradley, R. H. (2003). *HOME Inventory Administration Manual. Comprehensive Edition.* (U. of Arkansas, Ed.). Print Design, Little Rock.

Cavallera, V., Black, M., Bromley, K., Cuartas, J., Eekhout, I., Fink, G., … Dua, T. (2019). The Global Scale for Early Development ( GSED ). *Early Childhood Matters*, 80–84.

Fernald, L. C. H., Prado, E., Kariger, P., & Raikes, A. (2017). *A Toolkit for Measuring Early Childhood Development in Low and Middle-Income Countries* (Working Paper No. 122031). Washington, DC. Retrieved from http://documents.worldbank.org/curated/en/384681513101293811/A-toolkit-for-measuring-early-childhood-development-in-low-and-middle-income-countries

Fernald, L. C., & Hidrobo, M. (2011). Effect of Ecuador’s cash transfer program (Bono de Desarrollo Humano) on child development in infants and toddlers: a randomized effectiveness trial. *Social Science and Medicine*, *72*(9), 1437–1446. https://doi.org/0.1016/j.socscimed.2011.03.005

Gladstone, M., Lancaster, G. A., Umar, E., Nyirenda, M., Kayira, E., Van, N. R., … Smyth, R. L. (2010). The Malawi Developmental Assessment Tool (MDAT): The Creation, Validation, and Reliability of a Tool to Assess Child Development in Rural African Settings, *7*(5). https://doi.org/10.1371/journal.pmed.1000273

Grantham-McGregor, S., Cheung, Y. B., Cueto, S., Glewwe, P., Richter, L., & Strupp, B. (2007). Developmental potential in the first 5 years for children in developing countries. *The Lancet*, *369*(9555), 60–70. https://doi.org/10.1016/S0140-6736(07)60032-4

Hamadani, J. D., Huda, S. N., Khatun, F., & Grantham-McGregor, S. M. (2006). Psychosocial stimulation improves the development of undernourished children in rural Bangladesh. *The Journal of Nutrition*, *136*(10), 2645–2652. https://doi.org/136/10/2645 [pii]

Macours, K., Schady, N., & Vakis, R. (2012). Cash Transfers, Behavioral Changes, and Cognitive Development in Early Childhood: Evidence from a Randomized Experiment. *American Economic Journal: Applied Economics*, *4*(2), 247–273. https://doi.org/doi http://dx.doi.org/10.1257/app.4.2.247.

McCoy, D. C., Black, M. M., Daelmans, B., & Dua, T. (2016). Measuring development in children from birth to age 3 at population level. *Early Childhood Matters*, 34–39.

MICS-ECDI. (n.d.). MICS UNICEF. Retrieved from http://mics.unicef.org/

Nahar, B., Hamadani, J. D., Ahmed, T., Tofail, F., Rahman, a, Huda, S. N., & Grantham-McGregor, S. M. (2009). Effects of psychosocial stimulation on growth and development of severely malnourished children in a nutrition unit in Bangladesh. *European Journal of Clinical Nutrition*, *63*(6), 725–731. https://doi.org/10.1038/ejcn.2008.44

Rubio-Codina, M., Araujo, M. C., Attanasio, O., & Grantham-McGregor, S. (2016). *Concurrent Validity and Feasibility of Short Tests Currently Used to Measure Early Childhood Development in Large Scale Studies: Methodology and Results* (No. IDB-WP-723). *IDB-WP-723*. Washington, D.C. https://doi.org/10.1371/journal.pone.0160962

Rubio-Codina, M., Araujo, M. C., Attanasio, O., Muñoz, P., & Grantham-McGregor, S. (2016). Concurrent Validity and Feasibility of Short Tests Currently Used to Measure Early Childhood Development in Large Scale Studies. *Plos One*, *11*(8), e0160962. https://doi.org/10.1371/journal.pone.0160962

Weber, A. M., Rubio-Codina, M., Walker, S. P., van Buuren, S., Eekhout, I., Grantham-McGregor, S. M., … Black, M. M. (2019). The D-score: a metric for interpreting the early development of infants and toddlers across global settings. *Mimeo*.
