# Supplementary material for: Predictive validity in middle childhood of short tests of early childhood development used in large scale studies compared to the Bayley-III, the Family Care Indicators, height-for-age, and stunting: A longitudinal study in Bogota, Colombia
Source: PLoS One. 2020 Apr 29;15(4):e0231317. doi: 10.1371/journal.pone.0231317 (PMC7190101; doi:10.1371/journal.pone.0231317)
Supplement: Attachment — Submitted filename: Response to Reviewers.docx [file pone.0231317.s009.docx]

**PONE-D-19-20029R2**

**Predictive validity in middle childhood of short tests of early childhood development used in large scale studies compared to the Bayley-III, the Family Care Indicators, height-for-age, and stunting: A longitudinal study in Bogota, Colombia**

**Responses from the authors to the Reviewer’s comments**

We thank Reviewer 1 for reading through our second set of responses in detail. We respond below to the new set of comments. For clarity we retain the comments and add our response in *italics.* We have numbered the reviewers’ comments to facilitate cross-references to our responses. Please note that the references to the line numbers where edits have been made correspond to the version of the Manuscript without tracked changes (clean version or *Manuscript*).

**Reviewer Comments to the Author**

**Reviewer #1: PONE-D-19-20029R2
Predictive validity in middle childhood of short tests of early childhood development used in large scale studies compared to the Bayley-III, the Family Care Indicators, height-for-age, and stunting: A longitudinal study in Bogota, Colombia**

1. Overall, the value of the paper is in demonstrating predictive validity of various measures of child development. The data addressing this goal make a novel contribution. It is less valuable in guiding researchers’ selection of a measure. This is because the current data are limited in various ways: 1) they are from urban Colombia and unlikely to generalize to rural contexts or to Africa or Asia; 2) actual data on the short tests are presented in internally standardized form and so uninterpretable to other researchers; 3) the short tests/screeners had to be modified to prevent ceiling effects and so are inaccessible to other researchers; 4) the popular MDAT was not included and the GSED will shortly be the preferred measure. 5) I suggest that the aim of the current study be solely to provide predictive validity of the measures, though in the Discussion the authors could offer an opinion about use of the Denver if researchers need a short, easy-to-administer test in the near future.

*Response: The limitations mentioned by the reviewer were already acknowledged in the Manuscript and/or have been addressed in prior responses to comments from Reviewer 1. Specifically,*

1.1. *The need to use other tests/further investigation to be able to extrapolate findings outside urban Latin America was mentioned in lines 507-513: “Finally, the above findings could be extrapolated to urban Colombia and possibly urban areas in other Latin American countries. Further studies would be required before extrapolating to rural areas and other LMICs, especially in Africa and Asia, which could additionally evaluate more short tests for these contexts, such as the Malawi Developmental Assessment Tool (MDAT) (Gladstone et al., 2010).”*
1.2. *We recognize that the choice of internally or externally standardized scores is sometimes controversial. However, we disagree that the results would be “uninterpretable” because internally standardized scores were used. Internally standardized are the preferred scores in order to facilitate the comparison of performance of the various short tests, as indicated in lines 270-277: “No test had norms for Colombia. Therefore, we internally standardized the raw scores over age using age-conditional means and SDs, computed non-parametrically, after removing testers’/interviewers’ effects, as done in previous work [10]. This is, for each value of the residual of the raw scores on tester or interviewer dummies, we constructed a Z-score by subtracting the age-conditional mean and dividing by the age-conditional SD, both computed using local polynomial regressions.* *Unlike using norms from the reference populations (i.e. externally standardized scores) for each test, this standardization method handles age consistently across tests, which facilitates comparisons”. Lack of norms for tests is common in LMICs. In this situation, one may equally argue that using externally standardized scores in countries where the test is not standardized would be difficult to interpret.*

*Please note that all prior reviewers of this and the earlier paper investigating concurrent validity (Rubio-Codina, Araujo, Attanasio, Muñoz, & Grantham-McGregor, 2016), concurred with the use of the internally standardized scores as the best practice for the type of analysis we carry out.*

1.3. *It is not clear why these modifications are inaccessible to other researchers since they are explained in detail in previous work and in the Manuscript (see lines 172-180) “As reported earlier (Rubio-Codina, Araujo, Attanasio, Muñoz, et al., 2016), all tests were administered following manual instructions except the ASQ-3, which we modified as follows. A) Given the low literacy levels of some caregivers, caregiver-completed items were administered by interview in order to ensure all mothers understood the questions similarly. B) Furthermore, the child was tested if the caregiver did not know the answer. C) In addition, whenever the scale ceiling was reached, we added the next three non-overlapping items from subsequent questionnaires. This reduced the number of children on the test ceiling by 10.5–15.5% to levels of 1.7–4.8%, depending on the domain, thus increasing the variability in development captured by the test. Similar adaptations have been used elsewhere (Fernald, Kariger, Hidrobo, & Gertler, 2012).” It is worth highlighting the fact that these modifications have been used by other researchers in prior published work (Fernald et al., 2012).*
1.4. *The reasons why the MDAT was not included in the study have already been explained in prior responses and in the Manuscript—please see lines 511-513: “The number of short tests included in the current study was limited due to time and budgetary constraints. We therefore chose tests that were commonly used in large surveys and evaluations at the time the study began” and in the paragraph copied in point 1) above. We had already acknowledged that the GSED will be available in the future in Manuscript lines 48-50 “Efforts are currently underway to develop global (i.e. culturally-neutral or very easy-to-adapt), valid, feasible, freely accessible population-based instruments, as well as individual-level instruments suitable to evaluate interventions, for children 0-3 years* (Cavallera et al., 2019; McCoy, Black, Daelmans, & Dua, 2016)*.” However, the GSED will not be available immediately and these results can be helpful until then, as indicated in lines 61-64: “More generally, it is critical to identify readily available, reliable, valid and feasible ECD measures for use in large samples until the population-based and individual-level instruments currently under development become available and are shown to have both concurrent and predictive validity.” Moreover, the fact that the GSED is available does not necessarily imply that any of the prior existing tests are no longer used.*
1.5. *The aim of the study has been revised to highlight the study of predictive validity. However, since this can help guide the choice of tests, we have kept a mention to it and have rephrased lines 124-129 accordingly, which now read: “The study aimed to investigate the ability of the screeners (and other short tests and measures typically used as ECD proxies), given at three different age ranges, to predict later functioning across the developmental range, which can help to guide the choice of predictive, feasible and easy-to-use readily available instruments for the assessment of very young children’s development in large scale studies in LMICs, for either research purposes or as population-based indicators of ECD.”*

2. “Data” is a plural noun; verbs should accord.

*Response: We have revised the Manuscript for grammatical accuracy.*

3. Some figure and table titles use a preposition that does not make sense to me. E.g. S3 Fig “… validity of Bayley-III and short tests at 6-42 months of school achievement at 6-8 years…”. The preposition “of” seems incorrect, and maybe should be “with” or “and”. This occurs in several other tables/figures, e.g. line 351 and 363 “of FSIQ”.

*Response: Thanks. We have revised the Manuscript for grammatical accuracy.*

4. Line 51. I would delete the word “diagnostic” because none of the studies cited here is using the Bayley for diagnostic purposes; it is used as a research tool to assess child development. “Diagnosis” implies use of a test to identify problems needing treatment.

*Response: The word diagnostic is used to describe the Bayley-III, which is indeed a diagnostic test. We are not implying that the studies listed are being used to ‘diagnose’ children, however, we have removed the word so that there is no misunderstanding. And inserted “Whilst existing full developmental tests such as the Bayley Scales (…)”.*

5. Lines 52-54. Citation [2] referring to the WB toolkit is actually a citation to the Pearson description of the Bayley, and not derived from Fernald et al’s personal experience with the Bayley. I don’t mind that this be left unrevised, but it makes the authors appear naïve.

*Response: We have left the reference since the WB tookit concurs with these points: “administration of these tests requires a certain level of technical expertise and may be cumbersome for assessments that require a lot of manipulatives (e.g., Bayley Scales of Infant Development).” (Fernald, Prado, Kariger, & Raikes, 2017, p. 60). The authors of the toolkit also refer to time, cost, and copyright issues as limitations of the Bayley-III for administration in the context of program evaluations and hypothesis-driven research (see p. 65), as we had explained in our first set of responses to reviewers.*

6. Line 77. Who thinks that administering the Bayley at a center is the “ideal condition”? An unfamiliar center might make young children wary and distracted if they have not habituated to the surroundings.

*Response: A busy and noisy home with many interruptions may also distract the child. When comparing test scores, it is important that all children experience similar conditions as far as possible, that is the main reason for testing in centers because homes vary a lot and it is not possible to control interruptions. We have revised the sentence in lines 76-78, which now reads: “In contrast, the Bayley-III, which we considered our ‘gold standard’, was administered by psychologists at a center to minimize distractions and standardize the test experience as far as possible—and therefore, under preferable conditions.”*

7. Line 128. The authors responded to my concern about using these data to inform evaluations by saying that they agreed to delete such phrases. However, they need to delete the phrase “impact evaluations”. Possibly other data address the use of the Denver cognitive and language items in cash transfer, but the data from this study support its use for descriptive, not evaluative, designs.

*Response: Apologies for this omission. The words ‘impact evaluations’ have been deleted from line 129. Lines 123-129 now read: “**The study aimed to investigate the ability of the screeners (and other short tests and measures typically used as ECD proxies), given at three different age ranges, to predict later functioning across the developmental range, which can help to guide the choice of predictive, feasible and easy-to-use readily available instruments for the assessment of very young children’s development in large scale studies in LMICs, for either research purposes or as population-based indicators of ECD.”*

8. Line 489. I appreciate the paragraph on the choice of a measure being linked to its objective. However, the final statement about “further investigations” requires citations of existing research. There are already many studies using these measures to evaluate interventions and it is clear that many are not sensitive. The ASQ, for example, is frequently used and shows good effect sizes, but because mother-report is biased the findings are suspect.

*Response: We have listed references to evaluations in LAC, the context of relevance, that have used the Denver-II and the SFI-II, which are the two tests we would suggest using (in addition to the FCI). Please see lines 489-492: “Whilst both the Denver-II and the SFII have been found to be sensitive to the impact of cash transfer programs in Nicaragua (Macours, Schady, & Vakis, 2012) and Ecuador (Fernald & Hidrobo, 2011), respectively, further investigation of sensitivity to interventions for all short tests would be helpful in guiding the choice of tests. ” We are not suggesting researchers use any of the other tests, and less so the ASQ-3, as it shows very poor predictive validity (and had showed very poor concurrent validity in our previous work). Therefore, we do not see a need to provide references to those.*

9. It is surprising that no adaptations except translation and minor wording had to be made on the enrollment tests. Is this because the setting is urban Latin America? The stimulus booklet and picture book of the Bayley would need extensive adaptation in countries of Africa and Asia. So the authors’ claim to be searching for an easy-to-adapt measure has not been tested in places where adaptation would be more extensive.

*Response: We do not understand this point. Many of the enrollment tests had to be translated and pictures and wording had to be replaced and further modified after piloting. This was already explained in lines 199-204, in response to Reviewer 1, Comment 6 in our second set of revisions. These sentences read:* *“In preparation for testing, we translated and back-translated the Bayley-III; as well as the BDI-2 manual, and the WHO-Motor report forms and manual. Short tests in battery A were all* *available in Spanish. All translations and official Spanish versions were piloted and, subsequently, minor wording/phrasing modifications were made in order to reflect Colombian Spanish. Similarly, a few images had to be contextualized.* *Full adaptation details were provided earlier* (Rubio-Codina, Araujo, Attanasio, & Grantham-McGregor, 2016)*.” As explained in our prior revision, and owing to space restrictions and to avoid duplication with the previous publication, we did not add more adaptation details of the enrollment tests in the current manuscript. A link to the paper containing a complete description of the adaptation details was provided in the previous response document for the Reviewer’s reference and is also available here (see Appendix I in pages 40-42).*

10. The authors are unable to present anything other than internally standardized scores for tests other than the Bayley. The reason given is that they were screening tests and so required the addition of items and other major modifications. This is a major barrier to other researchers using those measures (the adapted measures with additional items would have to be obtained from these authors) and a major limitation in presenting findings to be compared with findings based on different measures of development. So the authors’ guidance on selection of a short measure is limited. Without comparable and interpretable data on the different short-form measures, researchers will not be able to use this study in their future work. The authors have selected the Denver in future work in China, but without interpretable evidence other researchers would have to take their word on faith.

*Response: We disagree this is a major barrier to other researchers as explained in our response to Comment 1.2 and 1.3 above. However, we are adding the raw scores of the short tests in Table S1.*

**References**

Cavallera, V., Black, M., Bromley, K., Cuartas, J., Eekhout, I., Fink, G., … Dua, T. (2019). The Global Scale for Early Development ( GSED ). *Early Childhood Matters*, 80–84.

Fernald, L. C. H., Kariger, P., Hidrobo, M., & Gertler, P. J. (2012). Socioeconomic gradients in child development in very young children: Evidence from India, Indonesia, Peru, and Senegal. *Proceedings of the National Academy of Sciences*, *109*(Supplement_2), 17273–17280. https://doi.org/10.1073/pnas.1121241109

Fernald, L. C., & Hidrobo, M. (2011). Effect of Ecuador’s cash transfer program (Bono de Desarrollo Humano) on child development in infants and toddlers: a randomized effectiveness trial. *Social Science and Medicine*, *72*(9), 1437–1446. https://doi.org/0.1016/j.socscimed.2011.03.005

Fernald, Lia C. H., Prado, E., Kariger, P., & Raikes, A. (2017). *A Toolkit for Measuring Early Childhood Development in Low and Middle-Income Countries* (Working Paper No. 122031). Washington, DC. Retrieved from http://documents.worldbank.org/curated/en/384681513101293811/A-toolkit-for-measuring-early-childhood-development-in-low-and-middle-income-countries

Gladstone, M., Lancaster, G. A., Umar, E., Nyirenda, M., Kayira, E., Van, N. R., … Smyth, R. L. (2010). The Malawi Developmental Assessment Tool (MDAT): The Creation, Validation, and Reliability of a Tool to Assess Child Development in Rural African Settings, *7*(5). https://doi.org/10.1371/journal.pmed.1000273

Macours, K., Schady, N., & Vakis, R. (2012). Cash Transfers, Behavioral Changes, and Cognitive Development in Early Childhood: Evidence from a Randomized Experiment. *American Economic Journal: Applied Economics*, *4*(2), 247–273. https://doi.org/doi http://dx.doi.org/10.1257/app.4.2.247.

McCoy, D. C., Black, M. M., Daelmans, B., & Dua, T. (2016). Measuring development in children from birth to age 3 at population level. *Early Childhood Matters*, 34–39.

Rubio-Codina, M., Araujo, M. C., Attanasio, O., & Grantham-McGregor, S. (2016). *Concurrent Validity and Feasibility of Short Tests Currently Used to Measure Early Childhood Development in Large Scale Studies: Methodology and Results* (No. IDB-WP-723). *IDB-WP-723*. Washington, D.C. https://doi.org/10.1371/journal.pone.0160962

Rubio-Codina, M., Araujo, M. C., Attanasio, O., Muñoz, P., & Grantham-McGregor, S. (2016). Concurrent Validity and Feasibility of Short Tests Currently Used to Measure Early Childhood Development in Large Scale Studies. *Plos One*, *11*(8), e0160962. https://doi.org/10.1371/journal.pone.0160962
