# Supplementary material for: Predictive validity in middle childhood of short tests of early childhood development used in large scale studies compared to the Bayley-III, the Family Care Indicators, height-for-age, and stunting: A longitudinal study in Bogota, Colombia
Source: PLoS One. 2020 Apr 29;15(4):e0231317. doi: 10.1371/journal.pone.0231317 (PMC7190101; doi:10.1371/journal.pone.0231317)
Supplement: Attachment — Submitted filename: Response to Reviewers.docx [file pone.0231317.s010.docx]

PONE-D-19-20029R3

**Predictive validity in middle childhood of short tests of early childhood development used in large scale studies compared to the Bayley-III, the Family Care Indicators, height-for-age, and stunting: A longitudinal study in Bogota, Colombia**

**Responses from the authors to the Reviewer’s comments**

We thank Reviewer 1 for reading through our third set of responses. We respond below to the new set of comments. For clarity, we retain the reviewer’s comments and number them to facilitate cross-references to our responses. Any changes to the text in the Manuscript are copied below in *italics.* Please note that the references to the line numbers where edits have been made correspond to the version of the Manuscript without tracked changes (clean version of the Manuscript).

**Reviewer Comments to the Author**

**Reviewer #1: PONE-D-19-20029R3
Predictive validity in middle childhood of short tests of early childhood development used in large scale studies compared to the Bayley-III, the Family Care Indicators, height-for-age, and stunting: A longitudinal study in Bogota, Colombia**

The authors have made some of the requested revisions and resisted others. I suppose some of the difference is between a statistical preference for drawing conclusions and giving advice based on the statistical analyses, and a preference for interpreting the methods and analyses in terms of child development within the context of an intervention.

So we can agree to disagree on whether it is better to test a child in his/her home where they have habituated to the "distractions" or to test in a controlled strange environment. Maybe you have to know about the "strange situation" to understand the difference.

We can also agree to disagree on whether Fernald was expressing an expert opinion on the Bayley or simply writing what Pearson Assessment has on their materials.

1. However, my statement that the authors should not state as an aim that they would provide guidance on which short measure to use was not sufficiently addressed. My point was not that you failed to mention the limitations, but that the limitations were sufficiently severe as to reduce the value of your advice on selection of a measure for others' research.

**Response:** We have omitted the part of the aim that claimed to provide guidance for the choice of instruments for the assessment of very young children’s development in large scale studies. The revised study aim, in lines 126-129 now reads: “*The study aimed to investigate the ability of the screeners and other short tests and measures, frequently used to evaluate interventions or measure child development at population level, given at three different age ranges, to predict later functioning in intelligence and school attainment.*”

Similarly, in the Discussion, we have deleted the reference to providing guidance for the choice of tests in lines 496-499, which now read: “*Whilst both the Denver-II and the SFII have been found to be sensitive to the impact of cash transfer programs in Nicaragua [12] and Ecuador [13], respectively, further investigation of sensitivity to interventions for all short tests would be helpful.*”

2. I understand that internally standardized scores make for good statistical comparisons across measures but they do not provide transparent information about the children's mental development using these measures. This study could be more than an exercise in statistical comparison if you want to provide guidance on selection of measures. The request for actual scores was answered by providing a supplementary table. The Denver scores were provided and I can see that they are aligned with the Bayley's in a ways that are similar to other papers and so are more transparent.

**Response:** We are pleased that this is now acceptable.

3. I can understand that other members of your team (e.g. Fernald) and other co-authors might be able to follow your non-standardized instructions to avoid ceiling on screening measures, but for obvious reasons other researchers will/have not been able to. Consequently, while your predictive data might be rigorous, others using these measures do not have the required adaptations to yield such valid data.

**Response:** We are sorry that the description of the modifications to the ASQ-3 were not clear. It was in fact similar to that used in the previous publication. We have modified the description in lines 174-184 to further clarify it and piloted it with colleagues and hope it is now clear: “*As reported earlier [10], all tests were administered following manual instructions except the ASQ-3, which we modified as follows. Given the low literacy levels of some caregivers, caregiver-completed items were administered by interview in order to ensure all mothers understood the questions similarly. Furthermore, the child was tested if the caregiver did not know the answer. In addition, whenever the scale ceiling was reached in the appropriate questionnaire for the age of the child, we added the next three more difficult items from the subsequent questionnaire excluding items that already occurred in the age-appropriate test. This reduced the number of children on the test ceiling by 10.5–15.5% to levels of 1.7–4.8%, depending on the domain, thus increasing the variability in development captured by the test. Similar adaptations have been used elsewhere [11].*”

4. I understand why you did not include the MDAT or refer to the GSED, but that does not eliminate the limitation that you advise people to use the Denver while the MDAT may have been a better test.

**Response:** We were saying that the Denver-II was the most appropriate out of the tests we examined—not of all available tests. We have made this clearer throughout the text:

Lines 473-476 in the Discussion: “*Over age 18 months, of the tests investigated, the Denver-II appeared to be the best candidate for use at scale, showing the closest predictive ability to the Bayley-III, although it was low-to-moderate, as indicated earlier.”*

Lines 553-554 in the Conclusion: “*The SFII from 19-30 months and the Denver-II from 19-42 months were the most feasible and valid short tests of those investigated.*”

Also, we were only suggesting that the Denver-II could be used in urban areas in Latin America, as indicated in lines 515-516: “*Finally, the above findings could be extrapolated to urban Colombia and possibly urban areas in other Latin American countries.*”

Subsequent to the publication of our previous paper on the concurrent validity of these tests, another study has shown that the Denver-II was also appropriate for Brazil so we have referenced this in lines 516-517: “*Following our earlier study of these tests’ concurrent validity [10], it was shown that the Denver was also appropriate for Brazil [55].*”

5. The authors left unchanged their aim as: "The study aimed to investigate the ability of the screeners (and other short tests and measures typically used as ECD proxies), given at three different age ranges, to predict later functioning across the developmental range, which can help to guide the choice of predictive, feasible and easy-to-use readily available instruments for the assessment of very young children’s development in large scale studies in LMICs, for either research purposes or as population-based indicators of ECD." I offered a compromise which the authors were unwilling to accept. I suggested that they not state as an aim that they would "guide the choice of predictive, feasible and easy-to-use readily available instruments …. for research purposes or as population-based indicators…" but rather provide their pros and cons in the Discussion to help guide researchers.

**Response**: Please see our response to Comment 1 above. As explained, we have dropped this aim

6. At the same time, in the Discussion they should also mention the untested option of the MDAT and the future D-score.

**Response**: Thanks for the suggestion. We have now mentioned several tests that could be evaluated in future including the MDAT and the GSED in lines 519-525: “*The number of short tests included in the current study was limited due to time and budgetary constraints. Short tests likely to be more suitable for Africa and Asia, such as the Malawi Developmental Assessment Tool (MDAT) [56] could be similarly evaluated. Moreover, several tests are currently under development including population-based and individual-level instruments for children 0-36 months [3,4] using a new approach, the D-score, which summarizes overall development using one interval scale [57]. After further piloting, these may be appropriate for use globally [4].* ”

7. They could also inform researchers that the Denver II is now 27 years old and there might be a new version coming out soon.

**Response:** Since the designer of the test has died, at present, there are no plans for a new version, but the manual and record forms are available for the publisher’s website (<http://denverii.com/>), so we are not mentioning this.

8. If researchers wanted a "guide to the choice of" good measures for their purpose, they would go to the World Bank Toolkit and its Inventory, not to a single study conducted in Bogota. For all these reasons and those mentioned initially (1.2, 1.3, 1.4), I recommend that the authors not state as an aim that they guide researchers' selection of a measure for research. Their Method is simply too limited to do this properly or convincingly. They can address this in the Discussion.

**Response**: We have dropped the aim. Please see our response to Comment 1 above. We have also added to the conclusions, the following (lines 559-560): “*These findings need to be examined in other regions, when recently developed tests, could be included.*”
